# Phylogenetic diversity of *Rhizobium* species recovered from nodules of common beans (*Phaseolus vulgaris* L.) in fields in Uganda: *R. phaseoli, R. etli*, and *R. hidalgonense*

**DOI:** 10.1093/femsec/fiae120

**Published:** 2024-09-12

**Authors:** Aregu Amsalu Aserse, Jean Nimusiima, John Baptist Tumuhairwe, Markku Yli-Halla, Kristina Lindström

**Affiliations:** Ecosystems and Environmental Research programme, Faculty of Biological and Environmental Sciences, University of Helsinki, Viikinkaari 1, P.O. Box 65, Helsinki, Finland; Ecosystems and Environmental Research programme, Faculty of Biological and Environmental Sciences, University of Helsinki, Viikinkaari 1, P.O. Box 65, Helsinki, Finland; College of Agricultural and Environmental Sciences, Department of Agricultural Production, Makerere University, P.O. Box 7062 Kampala, Uganda; College of Agricultural and Environmental Sciences, Department of Agricultural Production, Makerere University, P.O. Box 7062 Kampala, Uganda; Department of Agricultural Sciences, University of Helsinki, FIN-00014 Helsinki, Finland; Ecosystems and Environmental Research programme, Faculty of Biological and Environmental Sciences, University of Helsinki, Viikinkaari 1, P.O. Box 65, Helsinki, Finland

**Keywords:** biological nitrogen fixation, housekeeping genes, multi-locus sequence analysis, *Rhizobium*, symbiotic genes, symbiovar phaseoli

## Abstract

A total of 75 bacterial isolates were obtained from nodules of beans cultivated across 10 sites in six agro-ecological zones in Uganda. Using *recA* gene sequence analysis, 66 isolates were identified as members of the genus *Rhizobium*, while 9 were related to *Agrobacterium* species. In the *recA* gene tree, most *Rhizobium* strains were classified into five recognized species. Phylogenetic analysis based on six concatenated sequences (r*ecA–rpoB–dnaK–glnII–gyrB–atpD*) placed 32 representative strains into five distinct *Rhizobium* species, consistent with the species groups observed in the *recA* gene tree: *R. phaseoli, R. etli, R. hidalgonense, R. ecuadorense*, and *R. sophoriradicis*, with the first three being the predominant. The rhizobial strains grouped into three *nodC* subclades within the symbiovar *phaseoli* clade, encompassing strains from distinct phylogenetic groups. This pattern reflects the conservation of symbiotic genes, likely acquired through horizontal gene transfer among diverse rhizobial species. The 32 representative strains formed symbiotic relationships with host beans, while the *Agrobacterium* strains did not form nodules and lacked symbiotic genes. Multivariate analysis revealed that species distribution was influenced by the environmental factors of the sampling sites, emphasizing the need to consider these factors in future effectiveness studies to identify effective nitrogen-fixing strains for specific locations.

## Teaser

*Rhizobium* species were obtained from nodules of common bean plants in fields in Uganda.

## Introduction

Common bean (*Phaseolus vulgaris* L.) is an important food legume in eastern Africa. It serves as a primary dietary protein source for numerous households (Broughton et al. 2003) and source of income (Jjagwe et al. 2022). Uganda is the largest bean producer in Africa, and beans are ranked as the fifth most important crop in the country (Sibiko et al. 2013). Common bean cultivation is an integral component of the cropping systems in East Africa as beans are grown by 60%–90% of smallholder farmers (≤2 acres of land), and cultivated either as a sole crop or an intercrop with minimal inputs across all farming systems in Uganda. This practice partly enhances soil fertility but, more importantly, increases dietary diversity among smallholders (Muoni et al. 2019). In Uganda, beans are the most important food legume, followed by groundnut, soybean, and cowpea. The national annual consumption of beans in Uganda is around 58 kg per capita. Moreover, beans serve as a significant income source for many families, contributing up to 9% of household income in certain regions (Mazur et al. 2009).

Despite its significance, bean yields remain low, averaging 1.5 t ha^−1^ compared to potential yields of 4 to 5 t ha^−1^ (UBOS 2010, Kaizzi et al. 2012, FAO 2016). This yield gap is attributed to low soil fertility especially nitrogen (N) deficiency, and low abundance of N_2_-fixing bacteria in soils (Sebuwufu et al. 2015). In sub-Saharan Africa, soils are increasingly degraded, and fertilizers are often unaffordable for smallholders to enhance soil fertility (FAO 2016). Fertilizer use in Uganda is low, an average of 2.4 kg ha^−1^ yr^−1^ of NPK in 2021 (World bank, https://data.worldbank.org/indicator/AG.CON.FERT.ZS) yet the amounts of manure are insufficient to correct nutrient deficiencies. Therefore, finding ecologically sound and cost-effective methods to improve soil fertility is crucial for smallholder farmers. Food legumes, such as beans, form symbiotic associations with rhizobia (N_2_-fixing bacteria) to fulfill their demand for N, thus reducing the requirement for synthetic N fertilizer. Utilizing biological nitrogen fixation (BNF) through native or locally adapted rhizobia inoculants has bridged the yield gap in the production of bean and other food legumes (Kebede 2021).

Common beans form symbiotic relationships promiscuously with a diverse range of rhizobial species, particularly in the Mesoamerican and Andean centers of its diversification. Initially, *Rhizobium etli* was considered the dominant symbiont in these regions (Souza et al. 1997, Aguilar et al. 1998, Martínez-Romero 2003). However, other important bean-nodulating species have since been described, including *R. hidalgonense* (Yan et al. 2017), *R. esperanzae* (Cordeiro et al. 2017), *R. acidisoli* (Román-Ponce et al. 2016), *R. ecuadorense* (Ribeiro et al. 2015), *R. azibense* (Mnasri et al. 2014), *R. leucaenae* (Ribeiro et al. 2012), and *R. tropici* (Anyango et al. 1995). In regions where beans have been introduced, such as Europe and Africa, bean-nodulating species such as *R. phaseoli, R. gallicum, R. leguminosarum, R. lusitanum* (Dall'agnol et al. 2014), and *Pararhizobium giardinii* (Mousavi et al. 2015) are also prevalent. In Sub-Saharan African countries such as Ethiopia and Kenya, *R. etli* and *R. phaseoli* were predominantly isolated from bean nodules (Aserse et al. 2012, Mwenda et al. 2018, Gunnabo et al. 2021). Putative new species have also been identified in the region, including *R. ethiopicum*, a bean-nodulating rhizobia species in Ethiopia (Aserse et al. 2017)

The adaptability and diversity of bean-nodulating species vary depending on their origins, and different bean cultivars may prefer specific symbiotic partners (Aguilar et al. 2004). Previous studies conducted in Ethiopia, Uganda, Rwanda, and Kenya have shown mixed results regarding the response of beans to inoculation with exotic *R. tropici* CIAT 899 (Musandu and Joshua 2001, Simiyu et al. 2013, Rurangwa et al. 2018). While higher grain yields of beans in some areas were reported for inoculation with rhizobia compared to uninoculated beans, the response was not consistently positive. This erratic response may be attributed to the failure of exotic rhizobia to adapt to local conditions, influenced by environmental variables (Zhang et al. 2018). Locally isolated rhizobia are often better adapted to prevailing ecological conditions. Therefore, this study focuses on isolating and characterizing indigenous root nodule bacteria from Uganda as part of our efforts to find bean inoculants tailored to local conditions.

The taxonomy of bacteria has advanced with the use of molecular methods, such as protein coding gene sequencing data and whole-genome sequencing, which provide better resolution between species (Mousavi et al. 2015, Aserse et al. 2017). The gene coding for the recombinase A protein (*recA*) has been shown to be an effective genetic marker for rhizobia identification studies (Aserse et al. 2012, Mousavi et al. 2015, Asfaw et al. 2020, Adjei et al. 2022). However, phylogeny based on a single gene tree may not accurately represent a species tree, as housekeeping genes may vary in their evolutionary history. Phylogeny using multilocus housekeeping gene sequence analyses (MLSA), which include several housekeeping genes, provides more accurate resolution in the taxonomy of rhizobia species (Mousavi et al. 2015, Adjei et al. 2022).

This study aimed to explore the genetic diversity of *Rhizobium* species recovered from root nodules of common beans grown at ten major bean-growing locations across six agro-ecological zones in Uganda. Initially, bacterial isolates were identified through *recA* gene phylogenetic analysis. Subsequently, strains representing different species group were further studied using multilocus housekeeping genes and symbiotic gene sequences analyses. The MLSA included *recA*, glutamine synthetase II (*glnII*), RNA polymerase beta subunit (*rpoB*), DNA gyrase subunit B (*gyrB*), 70 kDa chaperone (*dnaK*), and ATP synthase subunit beta (*atpD*) coding genes. Phylogeny of the symbiotic genes was inferred using the analyses of *nodC* gene encoding *N*-acetylglucosaminyl transferase for nodulation and *nifH* that encodes dinitrogenase reductase. The nodulation capability of the representative strains was assessed in a growth chamber using beans as the host plant. We also examined how the physicochemical properties of sampling sites affect the taxonomic distribution and composition of the bacterial species identified in this study.

## Materials and methods

### Nodule sampling, bacteria isolation, and description of sampling sites

**Figure 1.**
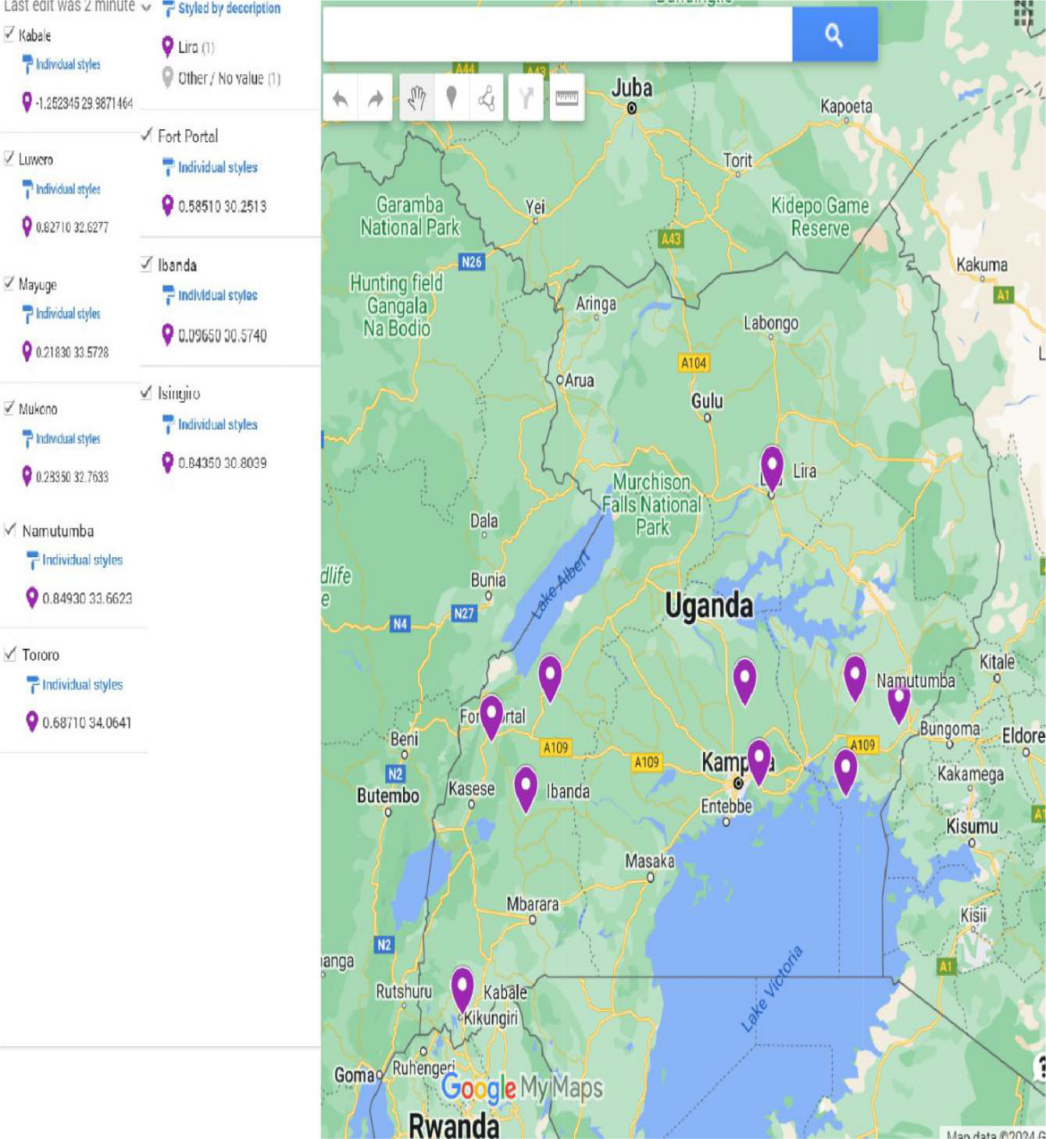
Soil and root nodule sampling sites in Uganda.

Root nodule samples were collected from common bean plants (cultivar Gambale Short) at 10 selected sites in major bean-growing regions, representing six agro-ecological zones in Uganda (Fig. 1). Several healthy and intact nodules were harvested per plant per site and stored in glass vials containing desiccant silica gel until isolation in the laboratory at Makerere University. Bacterial isolation was performed following the methodology described by Somasegaran and Hoben (2012). In brief, sterilized individual nodules were crushed, and their suspensions were streaked onto yeast extract mannitol (YEM) agar plates supplemented with 25 mg l^−1^ Congo red (CR). The plates were incubated at 28°C for 3–5 days or until colonies appeared. The resulting colonies, were further screened until pure culture was obtained on YEM agar medium at the University of Helsinki. Pure cultures of the isolates were then maintained and preserved in 20% glycerol–YEM broth at temperatures of −20°C and −80°C, as previously described (Aserse et al. 2012).

The agro-ecological zone, rainfall, and soil physicochemical properties of the sampling sites are presented in Table 1. Agro-ecological zones in Uganda were delineated by considering various characteristics, such as landscape, altitude, precipitation, climate, soil properties, demographics, and land use systems. These zones are key determinants of the country’s farming systems (MAAIF 2010). A summary of the sampled agro-ecological zones and their characteristics is also provided in Table S1. The annual rainfall of the sampling sites ranged from 1018 to 1482 mm. Soils were collected following procedures described previously (Andorson and Ingram 1993). The soil samples were analyzed for physico-chemical characteristics mainly according to van Reeuwijk (2002). Soil pH and EC were measured in 1:2:5 soil/water (v/v) suspension and the EC value was multiplied by 2.5 to get an EC estimate of saturated paste extract. Total C and N were determined by dry combustion, while soluble P was extracted by the Olsen method (0.5 M NaHCO_3_ at pH 8.5). Exchangeable Ca, Mg, K, Na, and titratable acidity were extracted with 1 M ammonium acetate buffered at pH 7, and results expressed as cmol(+) kg^−1^. Potential cation exchange capacity (CEC_pot_) and base saturation (BS) were calculated as CEC_pot_ = Ca+Mg+K+Na+titratable acidity and BS = 100 × (Ca+Mg+K+Na)/CEC_pot_, respectively. Exchangeable Al was extracted with 2 M KCl. The cationic elements were determined by inductively coupled plasma emission spectrometer and P with a spectrophotometer. Soil colors were determined with the Munsell Soil Color Charts. Texture was estimated by finger assessment.

**Table 1.** Soil properties of the sampling sites.

Site	Agro-ecological zone	Rain fall (mm yr^−1^)	Soil color code	Soil texture	C_tot_	N_tot_	BS	pH (H_2_O)	EC (dS m^−1^)	Ca	K	Mg	Na	TA	CEC_pot_	Al	P Olsen	
					(%)					(cmol_(+)_ kg^−1^)							(mg kg^−1^)	P class
Lira	Northern moist farmland	1218	7.5YR 4/2	cl	1.10	0.09	70	5.32	0.40	3.02	0.36	0.85	0.01	1.83	6.08	0.16	1.6	V low
Namutumba	Lake Victoria crescent	1317	5YR 4/4	fs	1.56	0.12	100	6.57	0.34	8.37	0.64	1.57	0.00	0.00	10.58	0.07	2.5	V low
Mayuge		1317	5YR 4/6	scl	0.78	0.07	100	5.04	0.34	1.43	0.31	0.51	0.02	0.00	2.28	0.33	7.8	Low
Luwero		1254	5YR–4/2	sl	2.65	0.14	100	7.72	0.72	27.41	1.75	2.51	0.13	0.00	31.80	0.06	105	V high
Mukono		1390	7.5YR–4/3	cl	2.14	0.16	100	7.36	0.36	10.62	0.97	2.34	0.03	0.00	13.95	0.08	35.6	V high
Kabale	Southwestern highlands	1018	10YR 5/4	sil	2.06	0.19	76	5.46	0.84	6.75	1.03	1.75	0.04	2.98	12.56	0.07	48.7	V high
Ibanda	Southwestern farmlands	1100	10YR 4/4	vfs	1.53	0.13	100	7.21	0.50	7.59	1.19	2.54	0.04	0.00	11.36	0.08	20.7	High
Isingiro		1063	7.5YR 5/4	sl	1.36	0.11	100	8.17	0.83	12.57	2.25	3.06	0.10	0.00	17.99	0.05	45.7	V high
Fort portal	Western medium-highland farmlands	1482	10YR 2/1	scl	4.57	0.35	93	6.95	0.41	20.76	1.76	4.85	0.07	2.19	29.62	0.05	183	V high
Tororo	Mount Elgon Farmlands	1468	10YR 4/4	sl	0.65	0.05	48	4.81	0.24	1.33	0.18	0.28	0.00	1.96	3.75	0.66	7.9	Low

C_tot_: total carbon organic carbon, N_tot_: total nitrogen.

cl = clay loam. sil = silt loam. sl = sandy loam. scl = sandy clay loam. vfs = very fine sand. fs = fine sand.

10YR–4/4 = Dark yellowish brown. 5YR–4/6 = Yellowish red. 7.5YR–5/4 = Brown. 10YR–4/4 = Dark yellowish brown. 10YR–5/4 = Yellowish brown.

10YR–2/1 = Black. 5YR–4/4 = Reddish brown. 7.5YR–4/3 = Brown. =7.5YR–4/2 = Brown. 5YR–4/2 =Dark brown gray

Results of Olsen P were interpreted according to Cottenie (1980).

The soils of the sampling sites were diverse in many characteristics. The texture ranged from clay loam to fine sand and total C from 0.7% to 4.6%. The CEC_pot_ ranging from 2.3 to 31.8 cmol_c_ kg^−1^, most results <18 cmol_c_ kg^−1^, suggests that many of the soils were dominated by kaolinitic mineralogy. The pH (H_2_O) covered a wide range, from 4.8 to 8.2. The Isingiro soil with the highest pH may contain some calcareous material but according to the C analysis the content is not high. All soils were non-saline, and the Na saturation was ≤1%. The two most acidic soils from Tororo and Mayuge sites had an appreciable Al saturation of 17 and 14%, respectively, while in the other soils, exchangeable Al occupied <3% of the CEC sites. The variation of plant-available P covered the whole range from very low to excessively high concentrations, suggesting that some of the soils had received large amounts of manure or chemical P fertilizers. Soil colour was brown or red in most soils, in accordance with the general abundance of Ferralsol (FAO 2014), which dominates about 70% of Uganda in the region, while the black colour of Fort portal soil falling in the order of Andisol, which covers much of the western region of Uganda.

### DNA extraction, PCR amplification, and sequencing

Genomic DNA was extracted from bacterial cultures grown for 3–5 days at 28°C in YEM until late log phase using the NucleoSpin Geomic DNA extraction kit, following the manufacturer’s instruction (MACHEREY-NAGEL, Inc.). The quality of the DNA samples was assessed using 1.5% (w/v) agarose gel electrophoresis and then stored at −20°C. For PCR amplification of the housekeeping gene *recA*, we used the primer pairs *recA*-6F (CGK CTS GTA GAG GAY AAA TCG GTG) and *recA*-555R (GACGR ATC TGG TTG ATG AAG ATC ACC AT) (Gaunt et al. 2001). The PCR was conducted using Phusion DNA polymerase following the manufacturer’s instructions (Finnzymes) for reaction mixture and temperature programs, and according to the protocols established in previous studies (Aserse et al. 2012, Asfaw et al. 2020). Subsequently, the *recA* PCR products was sequenced using the Sanger method at the Institute of Biotechnology, University of Helsinki.

### Sequence data analyses

The *recA* sequences were processed and edited using the Gap 4 program of the Staden-package 1.7.0 (Staden et al. 2000) or Mega11 software (Tamura et al. 2021). Sequences of closely related references to our test strains were identified using the Nucleotide Basic Local Alignment Search Tool (BLASTn) program (https://blast.ncbi.nlm.nih.gov/). The sequences alignments of the test and reference strains retrieved from GenBank were then aligned using CLUSTALW as embedded in MEGA 11 software. We employed the RAxML v8.2.X program to construct RAxML Phylogenetic tree using the GTRGAMMA model with rapid bootstrapping and codon position partitioning (Stamatakis 2014). For the RAxML phylogenetic tree, the robustness of the tree topologies was assessed using 100 bootstrap replicates. The pairwise *recA* sequence similarity between the new and reference strains was calculated using the BLASTn program (https://blast.ncbi.nlm.nih.gov/Blast.cgi?).

Based on the RAxML *recA* phylogenetic tree, 36 new strains representing different phylogenetic groups were selected and paired-end sequenced (2 × 150 bp) using the Illumina NextSeq500 platform. Additionally, we sequenced three Ethiopian bean-nodulating reference strains: *R. phaseoli* HBR10 and HBR53, and *R. etli* HBR5 (Aserse et al. 2012). Core housekeeping genes (*rpoB, dnaK, glnII, gyrB*, and *atpD*) and symbiotic (*nodC* and *nifH*) genes were then retrieved from the reads, as previously described (Adjei et al. 2022). The individual gene sequences of the test strains, along with relevant references retrieved from the GenBank database, were aligned using CLUSTALW. The six housekeeping genes were concatenated using Mega 11 software. Phylogenetic trees were constructed from the single gene alignments and the six concatenated housekeeping gene sequences, following a similar procedure as described above for the *recA* phylogenetic analysis. An RAxML phylogenetic tree based on combined gene sequences (*recA–rpoB–dnaK–glnII–gyrB–atpD*) was constructed using the GTRGAMMA model, with partitioning by DNA and codon positions, as described in the RAxML v8.2.X Manual (Stamatakis 2014).

**Figure 2.**
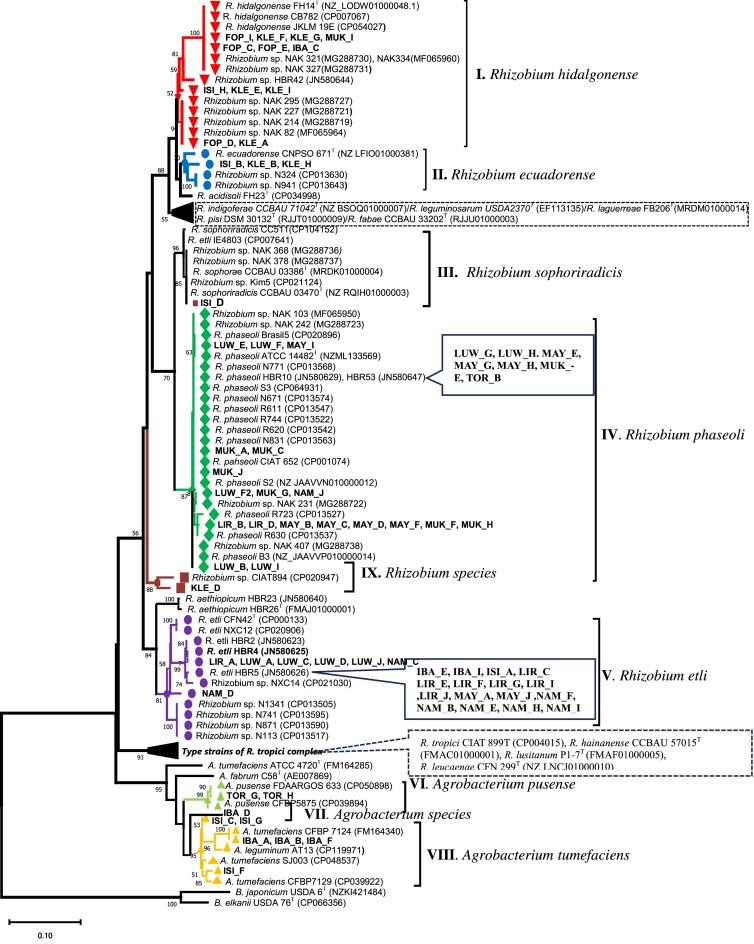
Maximum likelihood (RAxML) phylogeny based on *recA* gene (460 bp) sequences, depicting the taxonomic relationship between bacterial strains recovered from nodules of common beans in Ugandan (in bold) and closely related species of the genus *Rhizobium–Agrobacterium*. The phylogenetic tree was constructed using the GTRGAMMA model, with 100 bootstrap replications. Bootstrap values ≥50% are presented at the branch nodes. The scale bar, 0.01, indicates estimated nucleotide substitution rates. Gene sequence accession numbers of the references are in parenthesis, and type strains are indicated with superscript ‘T’. The sequence of *B. japonicum* USDA 6^T^ and USDA 76^T^ was included as an outgroup. B, *Bradyrhizobium*; R, *Rhizobium*; A, *Agrobacterium*.

The gene sequences for *recA, rpoB, dnaK, glnII, gyrB, atpD, nodC*, and *nifH* obtained in this study have been deposited in the GenBank database (https://www.ncbi.nlm.nih.gov/genbank/). Their respective accession numbers are as follows: *recA* (PP713623–PP713697), *rpoB* (PP713698–PP713733), *dnaK* (PP713770–PP713805), *glnII* (PP713734–PP713769), *gyrB* (PP713806–PP713841), *atpD* (PP713842–PP713877), *nodC* (PP732099–PP732133), and *nifH* (PP732134–PP732168). For the reference strains *R. phaseoli* HBR10 and HBR53, and *R. etli* HBR5, the accession numbers for the *dnaK, glnII, gyrB*, and *atpD* gene sequences are PP732090–PP732092, PP732087–PP732089, PP732093–PP732095, and PP732096–PP732098, respectively. The accession numbers of the reference sequences are indicated in parentheses in each single gene tree (Fig. 2; Figs S1–S5, Supporting Information).

### Nodulation authentication test

The nodulation ability of the 36 selected test strains, representing different phylogenetic groups in the RAxML *recA* phylogenetic tree, was assessed on the bean cultivar Gambale short under controlled growth chamber conditions, following the methods described by Adjei et al. (2022) and Aserse et al. (2012). The nodulation test was conducted in replicated pouches, with three seedlings transferred into each pouch containing sterile N-free Jensen’s nutrient solution (Somasegaran and Hoben 2012). Each seedling was inoculated with 1 ml of bacterial culture grown in YEM broth to log phase. After 30 days plant growth, the nodulation capacity of each strain was assessed based on the presence of nodules on the host roots. Preliminarily, the N₂-fixing ability of the bacterial strains was assessed based on the reddish to pink internal color of excised nodules and the healthy, green growth of the host plants.

### Effect of environmental variables on the distribution of the nodule bacterial species

To investigate the influence of environmental variables on the phylogenetic diversity (*recA*) of the test strains, Redundancy Analysis (RDA) was conducted. This analysis utilized the R program, following the methodology described by Adjei et al. (2022). Prior to analysis, explanatory variables (Table 1; Table S1, Supporting Information) were transformed (logX + 1) and normalized to address measurement unit heterogeneity (Clarke and Gorley 2001). Multivariate Analyses of Variance (MANOVA) were conducted to assess significant differences in environmental variables among sampling sites and *recA* phylogenetic groups. Pearson correlations between variable pairs were computed using the pearsonr() function from the scipy.stats package in Python. Additionally, a forward selection method in Python was employed to evaluate the separation power of variables among bacterial species. Redundant variables were systematically removed to mitigate multicollinearity effects. Finally, the resulting RDA was visualized using selected variables demonstrating significant separation power among the phylogenetic groups.

We assessed the diversity of the phylogenetic groups at each site using various diversity indices calculated with the vegan package in R. Species richness was determined using Shannon–Wiener diversity (H′) and True_Shannon indices. To evaluate species evenness and dominance, we employed the Simpson (D) and Simpson’s Index (λ), respectively. Additionally, Pielou’s evenness (J’) index was utilized to quantify the equity of species distribution across the sampling sites (Magurran 2004).

## Results

### Bacterial isolates, identification, and phylogeny based on single housekeeping genes

A total of 75 bacterial isolates were obtained from root nodules of common beans collected from 10 locations across six agro-ecological zones in Uganda (Table 1). Based on the comparison of the *recA* gene sequence (464 bp) with the GenBank reference database, 66 isolates were identified as belonging to the genus *Rhizobium*, while 9 isolates were classified as *Agrobacterium* species. The RAxML phylogenetic tree, constructed with appropriate reference sequences, grouped the strains into nine phylogenetic groups (Fig. 2). The main phylogenetic groups (I, II, IV, and V) comprised between three and 26 test strains each, alongside known common bean-nodulating reference *Rhizobium* species. These groups exhibited *recA* sequence similarity ranging from 96% to 100% with each other and with their respective closely related reference type strains (Table 2). In group I, 13 test strains were closely clustered with *R. hidalgonense* and bean-nodulating *Rhizobium* species from Kenya (Mwenda et al. 2018). Phylogenetic groups II (three strains), IV (26 strains), and V (23 strains) clustered with named *Rhizobium* species, specifically the type strains of *R. ecuadorense, R. phaseoli*, and *R. etli*, respectively. Additionally, the latter two groups included bean-nodulating rhizobial species obtained from Kenya (Mwenda et al. 2018) and Ethiopia (Aserse et al. 2012). The strains included in group I, IV, and V were distributed across five to seven sampling sites (Table S3, Supporting Information). Minor phylogenetic groups (III, VI, VII, VIII, IX) contained only one to three strains each. In group III, test strain ISI_D obtained from Isingiro sampling site clustered tightly with reference species *R. sophoriradicis* CCBAU 03470^T^, *R. sophorae* CCBAU 03386^T^, *R. sophoriradicis* CC511, *R. etli* IE4803, and Kenyan *Rhizobium* species. A single strain, KLE_D isolated from Kabale sampling site, in group IX shared 96% sequence similarity with *Rhizobium* strain CIAT894 obtained from Colombia (Acosta et al. 2011). Strains belonging to *Agrobacterium* species (groups VI–VIII) were exclusively obtained from Tororo, Isingiro and Ibanda sampling sites. Strains in groups VI and VIII showed >97% sequence similarity with *A. pusense* and *A. tumefaciens*, respectively. Group VII included only strain IBA_D without close references, sharing only 94% sequence similarity with *A. pusense* CFBP5875 or *A. tumefaciens* SJ003.

**Table 2.**
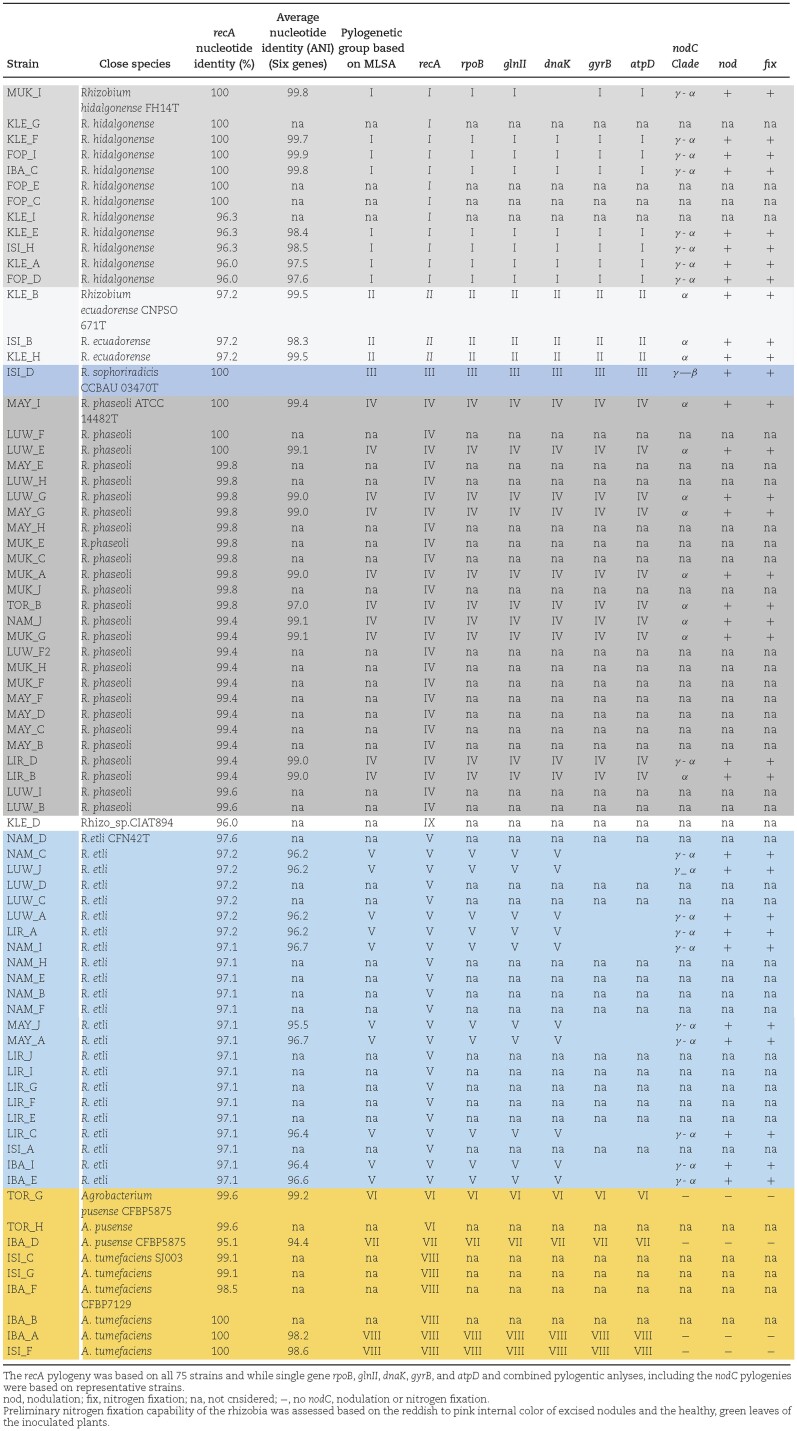
Taxonomic classification and species affiliation of strains based on concatenated housekeeping (MLSA) and single gene sequences analyses, and nodulation status identified in this study.

The phylogeny of 36 strains, representing different *recA* groups, was further examined through sequence analysis of *rpoB* (945 bp), *dnaK* (1013 bp), *glnII* (863 bp), *gyrB* (671 bp), and *atpD* (460 bp) genes. RAxML phylogeny of the individual housekeeping genes are depicted in Figs S1–S5 (Supporting Information). Despite slight topological variations, the groups identified in the *recA* phylogenetic tree (Fig. 2) were consistently recovered in all individual gene trees. Strains in groups I, II, III, IV, V, VI, and VIII exhibited >95% sequence similarity in all genes with their respective closest reference strains, as identified in the *recA* phylogenetic tree (Data not shown). However, strains NAM_C, LUW_J, LUW_A, and LIR_A, closely related to *R. etli* in the *recA* tree, formed a separate cluster in *gyrB* trees alongside unnamed bean *Rhizobium* species from Mexico (>97% sequence similarity). In the a*tpD* phylogenetic tree, these strains clustered with *R. aethiopicum* HBR26^T^, sharing >98% sequence similarity.

### Phylogeny based on concatenated multilocus sequence analysis

**Figure 3.**
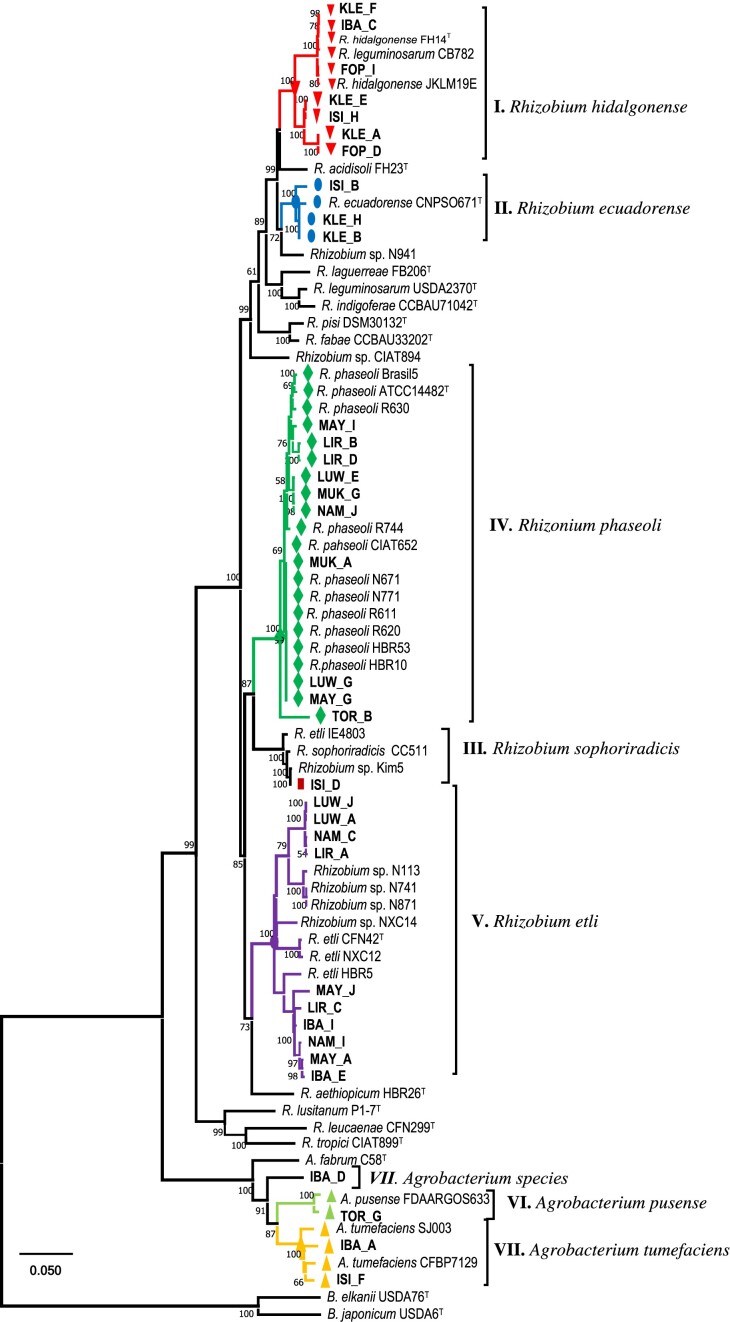
Maximum likelihood (RAxML) phylogeny based on based on *recA–rpoB–dnaK–glnII–gyrB–atpD* combined gene sequences (4416 bp), illustrating the taxonomic relationship among bacterial strains recovered from nodules of common beans in Ugandan (in bold) and closely related species of the genus *Rhizobium–Agrobacterium*. The phylogenetic tree was constructed using the GTRGAMMA model with partitioning of each DNA and codon positions, and with 100 bootstrap replications. Bootstrap values ≥50% are presented at the branch nodes. The scale bar, 0.05, indicates estimated nucleotide substitution rates. Gene sequence of the reference type strains are indicated with superscript ‘T’. The sequence of *B. japonicum* USDA 6^T^ and USDA^T^ was included as an outgroup. *Bradyrhizobium*; R, *Rhizobium*; A, *Agrobacterium*.

A concatenated phylogeny was constructed based on aligned sequences from the *recA* (464 bp), *rpoB* (945 bp), *dnaK* (1013), *glnII* (863 bp), *gyrB* (671 bp), and *atpD* (460 bp) genes to refine further the taxonomic position of the strains. The combined sequence spanning 4416 bp, comprised 281 conserved sites, 183 variable sites, 170 parsimony-informative sites, and 13 singleton sites (Table S2, Supporting Information). Several reference strains used in single gene trees were excluded from the combined analysis due to lack of *glnII, gyrB*, or *atpD* gene sequences. The topology of the RAxML concatenated phylogenetic tree (Fig. 3) closely resembled the individual gene trees but with higher bootstrap support values (BT).

In the phylogeny based on MLSA, the test strains were classified into five *Rhizobium* and three *Agrobacterium* monophyletic lineages (BT > 91%), consistent with the single gene phylogenies assigned as groups I–VIII (Fig. 2; Figs S1–S5, Supporting Information).

The taxonomic affiliation of the test strains is tabulated in Table 2. In group I, eight test strains and *R. hidalgonense* JKLM 19E and CB782 shared 97.6%–99.9% average nucleotide identity (ANI) of the combined sequences with the type strain *R. hidalgonense* FH14^T^. Within group II, strains exhibited 98.3%–99.5% ANI with the closest type strain *R. ecuadorense* CNPSO671^T^. Group III included a single test strain, ISI_D, which tightly clustered (BT = 100%) with *R. sophoriradicis* CC511, and *R. etli* IE4803 (99.1%–100% ANI). ISI_D also shared 100% nucleotide similarity with *R. sophoriradicis* CCBAU 03470^T^ across all single gene sequences, except for *glnII*, for which gene sequence was lacking for the type strain. Group IV contained eight test strains, sharing 97.0%–99.4% ANI with the closest type strain *R. phaseoli* ATCC 14482^T^. This group also included reference strains, such as *R. phaseoli* CIAT 652, and *R. phaseoli* Brasil5, along with other bean-nodulating *R. phaseoli* strains obtained from Mexico and Ethiopia (Aserse et al. 2012). Group V, comprised 10 test strains classified as *R. etli*, displayed ANI values ranging from 95.5% to 96.6% with the closest type strain *R. etli* CFN 42^T^. Intriguingly, the four strains that formed aberrant clusters in *gyrB* and *atpD* single gene phylogenies were also assigned to group V and conflicting signals were resolved in the combined tree with robust branch support value (BT = 100%). Strains belonging to the genus *Agrobacterium* were consistently classified in groups VI, VII and VIII, as observed in the single gene trees. The sole *Agrobacterium* strain, IBA_D, in group VII was unique, lacking close references and sharing only 94.2%–94.4% ANI with *A. tumefaciens* SJ003 and *A. tumefaciens* CFBP7129.

### Symbiotic gene phylogeny

**Figure 4.**
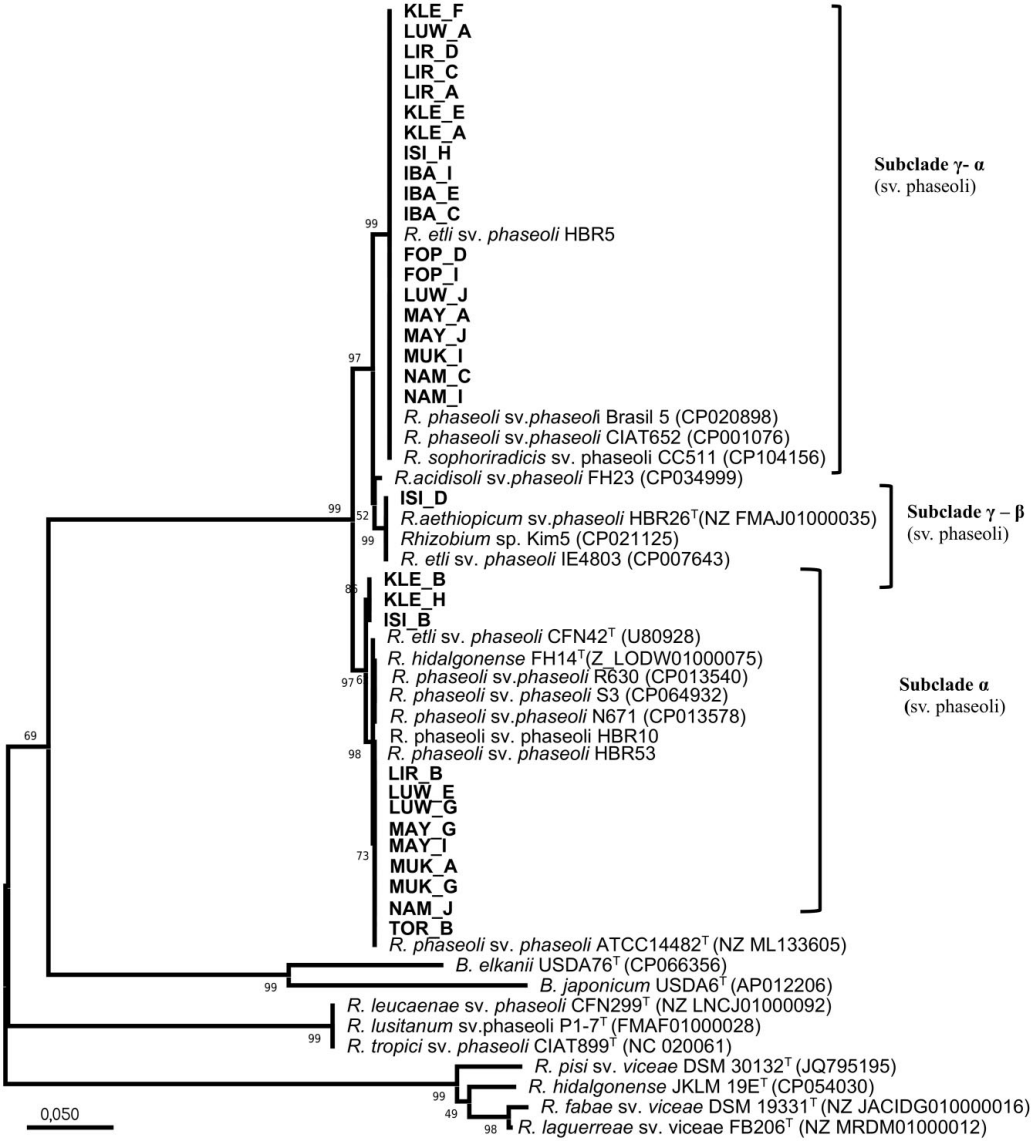
Maximum likelihood (RAxML) phylogeny based on *nodC* (1020 bp) gene sequences, showing symbiotic gene relationship between Ugandan common bean-nodulating rhizobial strains (in bold) and closely related species. Bootstrap values ≥50% are presented at the branch nodes. The scale bar, 0.05, indicates estimated nucleotide substitution rates. Gene sequence accession numbers of the references are in parenthesis, and type strains are indicated with superscript ‘T’. B, *Bradyrhizobium*; R, *Rhizobium*.

Symbiotic phylogeny was investigated through sequences analyses of the *nodC* (1020 bp) and *nifH* (651 bp) genes obtained for 32 representative strains. Strains belonging to *Agrobacterium* species did not possess *nodC* and *nifH* genes. The *nifH* gene exhibited high conservation, with the 32 test strains and bean-nodulating references clustering into a single clade symbiovar phaseoli, sharing nearly 100% sequence similarity in the *nifH* gene. Therefore, displaying the RAxML *nifH* phylogenetic tree was deemed unnecessary and excluded. In the RAxML *nodC* phylogenetic tree (Fig. 4), all representative *Rhizobium* strains were grouped into the clade symbiovar phaseoli, categorized into three subclades with 100% bootstrap support values, previously recognized as α, γ–α, and γ–β (Rouhrazi et al. 2016, Mwenda et al. 2018). The two major subclades in this study, α and γ–α, consist of strains belonging to different species. Strain ISI_D, in clade γ–β, shared 100% sequence similarity to the *nodC* of *R. aethiopicum* sv. *phaseoli* HBR26^T^ and *R. etli* sv. *phaseoli* IE4803. Clade α comprised 12 test strains belonged to *R. phaseoli* and *R. ecuadorense* (groups II, IV), sharing identical or nearly identical *nodC* sequence with the type strains *R. phaseoli* and *R. etli*, as well as other bean-nodulating *R. phaseoli* sv. p*haseoli* strains from neighbor Ethiopia (Aserse et al. 2012) and Kenya (Wekesa et al. 2023). Clade γ–α included 19 test strains belonging to *R. etli* (group V) and *R. hidalgonense* (group I), exhibiting 100% *nodC* sequence similarity with *R. etli* sv. *phaseoli* HBR5, *R. phaseoli* CIAT652 and Brasil5, and *R. sophoriradicis* sv. phaseoli CC511. Notably, the *nodC* phylogeny revealed that the strain *R. hidalgonense* JKLM 19E, contrary to its taxonomic position, closely clustered with symbiovar viciae species, such us *R. pisi* sv*. viceae* DSM 19331^T^ and *R. fabae* sv. *viceae* DSM 19331^T^. Overall, *nodC* and *nifH* phylogenies appeared incongruent and different from the MLSA phylogeny. Regarding symbiotic gene phylogeny, the bean-nodulating Ugandan strains showed limited diversity compared to their taxonomic diversity.

### Nodulation ability of the test strains

Of the 36 bacterial strains selected for nodulation test, all 32 strains belonging to *R. hidalgonense, R. ecuadorense, R. phaseoli, R. etli*, and *R. sophoriradicis* successfully induced pink nodules on the roots of the host bean plants, which appeared green and healthy. In contrast, four strains classified as *Agrobacterium* species failed to form symbiosis with the beans. Information regarding the nodulation status of the isolates is presented in Table 2.

### Effect of environmental variables on the distribution of identified phylogenetic groups

**Figure 5.**
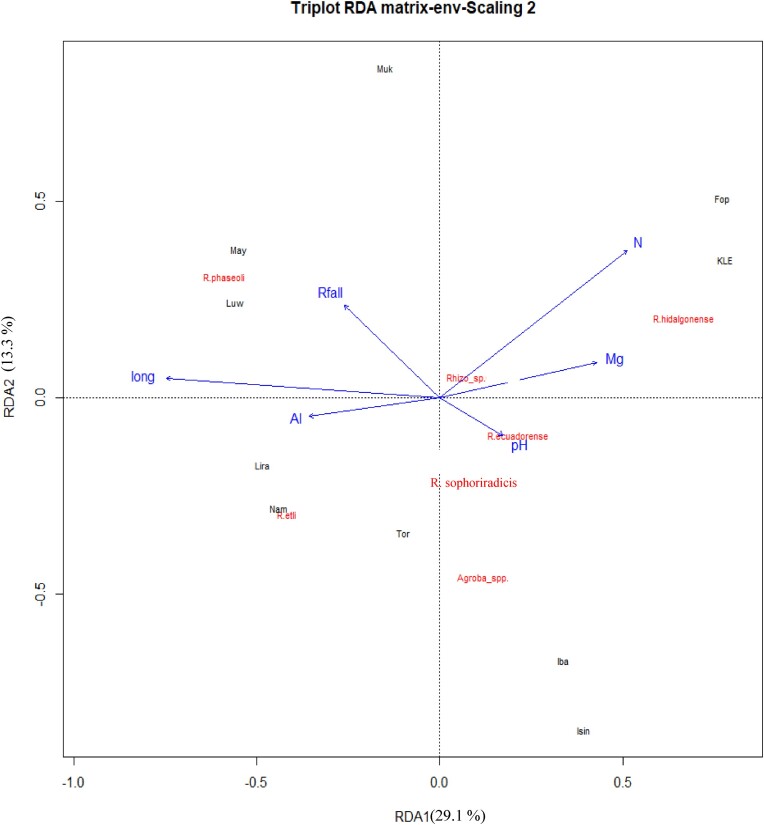
RDA Triplots illustrating the relationship among environmental variables (blue) and common bean root nodule bacterial species (red), classified by *recA* phylogeny. The first two RDA axes together explained 42.4% of the total variance. Rfall, rainfall; long, longitude; Al, exchangeable acidity; Mg, magnesium; N, nitrogen. short names correspond to the sampling sites listed in Table 1.

The distribution of identified phylogenetic groups was significantly influenced by environmental variables at the sampling sites, particularly with rainfall, soil pH and total N, and exchangeable Al and Mg. These variables exhibited higher separation power between the species groups categorized using *recA* phylogeny, indicating their importance in shaping the distribution of the test strains. MANOVA test results revealed significant differences (*P* < 0.005, data not shown) in these variables among *recA* phylogenetic groups (Fig. 2; Table 2). In the RDA triplot (Fig. 5), the first two axes collectively explained about 42% of the total variance and represented the environmental variables well. Increasing soil Mg and/or total N positively affected the distribution of strains related to *R. hidalgonense* (group I) but had a negative impact on the distribution of *R. phaseoli* (group IV) and *R. etli* (group V). Strains belonging to *R. hidalgonense* were notably abundant in Fort portal and Kabale sites, where soil pH ranged between 5.5 and 7, and the concentrations of Mg and total N were higher compared to other sites (Table 1). Additionally, these sites exhibited higher C content, suggesting that the abundance of these species is influenced by higher soil organic matter content correlating closely with total N (*r* = 0.93). Variables such as longitude, increasing rainfall, and exchangeable Al exhibited a positive influence on the distribution of strains classified under *R. phaseoli*. The distribution of strains related to *R. etli* appeared to correlate with an increase in exchangeable Al concentration to some extent. The distribution of strains belonging to *Agrobacterium* species seemed to be associated with higher soil pH. On the contrary, increasing soil pH had a negative effect on the distribution of *R. etli* species, suggesting that *R etli* seems to do well in acidic soils where the exchangeable Al concentration increases.

The species distribution across the sampling sites and diversity indices are summarized in Table S1 and Table S3, Supporting Information). Isingiro exhibited the highest species richness (Margalef), Shannon–Wiener Index (H'), Simpson’s Index (λ), and True-Shannon index values (2.06, 1.50, 0.73, 4.37), followed by Ibanda and Kabale, each site represented by five to three species, respectively. Fort Portal had the lowest richness, Shannon–Wiener (H'), Simpson’s Index (λ), and True Shannon (0.00), with only one species identified. Overall, our results emphasize the need for a larger, more evenly distributed sample size per site to improve the accuracy of diversity assessments.

## Discussion

In this study, 75 bacterial isolates were recovered from root nodules of bean plants collected from ten selected bean-growing locations in Uganda. Initial analysis of *recA* gene sequences classified 66 strains into six *Rhizobium* phylogenetic groups and nine into three *Agrobacterium* groups. MLSA using six housekeeping genes conducted for 36 selected strains representing the *recA* phylogenetic groups revealed the classification of most *Rhizobium* strains into *R. phaseoli, R. etli, R. hidalgonense*, and *R. ecuadorense*, with a few minor groups like *R. sophoriradicis* ISI_D and *Rhizobium* sp. KlE_D. Similarly, *Agrobacterium* strains were classified as *A. pusense, A. tumefaciens*, and *Agrobacterium* sp. The *Rhizobium* strains (32) fell within the symbiovar *phaseoli* clade and were further categorized into three subclades in the *nodC* phylogenetic tree. However, the strains showed high conservation in the *nifH* gene, with nearly identical sequences among themselves and references.

In previous studies (Miranda-Sánchez et al. 2016, Asfaw et al. 2020, Adjei et al. 2022), the *recA* gene has been used for classifying root nodule bacteria, with its phylogenetic groups generally aligning with MLSA-derived species trees. Still, MLSA, which utilizes multiple protein-coding housekeeping genes, remains the preferred method for accurately classifying closely related rhizobial species (Aserse et al. 2012, Mousavi et al. 2015, Adjei et al. 2022). In this study, strains belonging to a single species formed distinct *recA* phylogenetic groups, consistent with the species groups identified in the MLSA tree derived from six concatenated alignments. The *recA* gene sequence similarity between strains and the closest type species was >97%, and the combined six-gene nucleotide identity was >96%, with the latter aligning with the 96% threshold for species delineation proposed by Konstantinidis et al. (2006). The species groups were also consistent across rpoB, dnaK, glnII, gyrB, and atpD single-gene phylogenetic trees, although there were some aberrant classifications in the gyrB and atpD gene trees. These anomalies are likely due to gene-specific evolutionary histories or horizontal gene transfer (Aserse et al. 2012, Mousavi et al. 2015) but were resolved by the robust concatenated phylogenetic tree, which is supported by high bootstrap values (Fig. 3).

Beans are known to establish symbiosis with various rhizobial species, particularly in the Mesoamerican and Andean centers of bean diversification (Ribeiro et al. 2012, Tong et al. 2018). Nevertheless, rhizobia taxonomic species diversity can vary by region, local conditions, or bean host cultivar (Aguilar et al. 2004, Zhang et al. 2018). In this study, the identification of *R. phaseoli, R. etli, R. hidalgonense*, and *R. ecuadorense* from nodules of the bean cultivar Gambale Short highlights the taxonomic diversity of bean-nodulating rhizobia in Uganda. Among these species, *R. phaseoli* and *R. etli* are particularly widespread, commonly found in regions where beans have been introduced. Notably, *R. etli* has been found in bean seeds, potentially contributing to its global distribution (Perez-Ramirez et al. 1998, Rodino et al. 2010). This species also has a broad host range, forming nodules with other legumes, such as *Mimosa* and *Acacia* species. Interestingly, a study by Miranda-Sánchez et al. (2016) found that in Mexican soils, *R. etli* was more commonly associated with *Acacia* species than with beans when used as trapping hosts. The prevalence of *R. etli* and *R. phaseoli* species in Ugandan soils consistent with previous findings in the Sub-Saharan Africa countries, such as Ethiopia and Kenya (Aserse et al. 2012, Mwenda et al. 2018, Gunnabo et al. 2021). However, to the best of our knowledge, this is the first study to identify *R. hidalgonense* and *R. ecuadorense* as bean-nodulating species in the eastern African region, in Uganda. *R. ecuadorense* is an indigenous effective N_2_-fixing bean symbiont in Ecuador, isolated from various sites in country. Additionally, a representative strain was also obtained from Mexico (Ribeiro et al. 2015). Conversely, *R. hidalgonense*, including the type strain FH14^T^, was obtained from acidic soils in Hidalgo state in Mexico (Yan et al. 2017). In our study, strains belonging to this species were recovered from bean nodules grown in soils with a pH range of 5 to 8 from different locations. Based on *recA* and *atpD* gene phylogenesis, *R. hidalgonense* group also included some reference strains isolated from bean nodules in Kenya (Mwenda et al. 2018) and Ethiopia (Aserse et al. 2012) that were classified as undefined *Rhizobium* species, such as NAK334 and HBR4, respectively. These *Rhizobium* strains are likely to belong to *R. hidalgonense*, although their identities require further validation through additional gene sequence analyses.

Various non-symbiotic endophytic bacteria, including *Agrobacterium* species, are commonly found in legume nodules (Mhamdi et al. 2005, Aserse et al. 2012). These bacteria were found to colonize bean nodules only when co-inoculated with symbiotic rhizobia (Aserse et al. 2013). In our study, *Agrobacterium* strains lacked the symbiotic genes *nodC* and *nifH* and were unable to form nodules on host plants. These non-symbiotic strains likely coexisted with symbiotic bacteria in the same nodules, with their entry probably facilitated by the symbiotic bacteria (Aserse et al. 2013).

The symbiotic gene phylogeny provides insights into the symbiovar and host ranges of rhizobia species. While *nodA, B*, and *C* genes encode crucial components of the lipo-oligosaccharide core structure, Nod factor backbone, essential for rhizobial host infection (Roche et al. 1996), the *nifH* gene encodes the nitrogenase Fe protein, vital for N_2_ fixation (Hu et al. 2006). Despite their taxonomic divergence, Ugandan bean-nodulating species clustered into three subclades within symbiovar phaseoli (Fig. 4), all sharing nearly 100% *nifH* sequence identity. These subclades were also identified in bean-nodulating rhizobial species from Kenya and Iran (Rouhrazi et al. 2016, Mwenda et al. 2018). The main *nodC* alleles/subclades, α and γ- α, correspond to symbiovar Clade I and Clade II identified in Ethiopian bean-nodulating strains (Aserse et al. 2012). Phylogenies of symbiotic and housekeeping genes are often incongruent due to their independent evolutionary histories, as housekeeping genes are chromosomal and symbiotic genes are plasmid-borneor or located on other accessory elements. Horizontal gene transfer (HGT) is considered the main factor driving the evolution of symbiotic genes, enabling taxonomically distinct rhizobial species to share similar symbiotic genes (Laguerre et al. 1996, Rogel et al. 2011). This study found that the *nodC* subclades included strains with diverse chromosomal backgrounds from various countries (Rouhrazi et al. 2016, Mwenda et al. 2018), suggesting that symbiotic genes evolved independently and were likely acquired through HGT among bean-nodulating rhizobial species. We also observed a clustering pattern in which test strains classified as *R. phaseoli* and *R. ecuadorense* shared subclade α, while those belonging to *R. etli* and *R. hidalgonense* clustered in subclade γ–α sv. phaseoli, indicating the exchange of the same symbiotic genes through HGT among specific species groups. The clustering of *R. hidalgonense* FH14^T^ with subclade α of *symbiovar phaseoli* and *R. hidalgonense* JKLM 19E with symbiovar viciae species (*R. pisi* sv. v*iciae*), respectively, reveals that strains of the same species can exhibit different symbiovars and host ranges (Tong et al. 2018). Apparently, the latter strain was isolated from effective nodules of pea (*Pisum sativum* L.) from the Indian trans-Himalayas (Rahi et al. 2020).

The microbial community structure, distribution, composition, functions are intricately influenced by soil and environmental properties (Zhang et al. 2011). Soil factors are known to play a crucial role in bacterial nodule occupancy (de Castro Pires et al. 2018). The effects of soil properties, such as pH, particle size distribution, total N, exchangeable Ca and Al on rhizobia genospecies distribution have been well documented (Asfaw et al. 2020, Adjei et al. 2022). Consistent with these findings, our research identified soil pH, Al, Mg, N, and rainfall at sampling locations as key factors influencing the distribution of *Rhizobium* species obtained from nodules of the bean cultivar Gambale Short in Uganda (Fig. 5). Specifically, *R. phaseoli* and *R. etli* were predominantly found in the Lake Victoria and Northern moist farmland agro-ecological zones (sites like Namutumba, Mayuge, Luwero, Mukono, and/or Lira), exhibiting a positive correlation with increasing exchangeable Al, and rainfall. The soil in these sites was either acidic or circum neutral pH, and the Mg concentrations were generally low, likely due to leaching caused by high rainfall, a common characteristic of humid areas. Conversely, strains belonging to *R. hidalgonense, R. ecuadorense* phylogenetic groups, and/or *Agrobacterium* spp. were more commonly obtained in the Western medium-highland farmlands (Fort Portal), Southwestern farmland (Ibanda, Isingiro), or Southwestern highlands (Kabale) agro-ecological zones, where their distribution patterns appeared to be influenced by increasing soil N, Mg, and/or pH levels. The close correlation of soil N with soil C proposes that the distribution of these species is determined by soil organic matter content as well. The sampling site Isingiro, characterized by alkaline soil (pH 8.17) and lower rainfall, exhibited the highest species richness diversity index (Table 1; Table S3, Supporting Information). However, it’s essential to note the small and unevenly distributed sample size per site (4–12), which may impact the accuracy of diversity indices. Overall, the variation in composition and distribution patterns of *Rhizobium* species (Fig. 5; Table S3, Supporting Information) among the sampling sites reflects their adaptation to different environmental properties (Table 1). This underscores the need to isolate locally adapted *Rhizobium* species as inoculants, as their competitiveness for nodulation and N₂ fixation relies on their adaptability to local conditions.

## Conclusion

Based on nodule samples collected from ten key bean-growing regions across six agro-ecological zones in Uganda, we identified bean-nodulating rhizobia as *R. phaseoli, R. etli, R. hidalgonense*, and *R. ecuadorense*, all within the symbiovar phaseoli. The *Rhizobium* species distribution was influenced by the soil and environmental factors at the sampling sites. Nodulation tests with representative strains indicated that the *Rhizobium* species could form symbiosis with bean hosts. However, further evaluation of N₂ fixation under greenhouse and field conditions is essential for selecting effective N₂-fixing strains for bean inoculation, considering specific environmental factors to optimize N₂ fixation, enhance soil productivity, and improve bean yields.

## Supplementary Material

fiae120_Supplemental_Files

## Data Availability

The gene sequences for recA, rpoB, dnaK, glnII, gyrB, atpD, nodC, and nifH obtained in this study have been deposited in the GenBank database, https://www.ncbi.nlm.nih.gov/genbank/.

## References

[bib1] Acosta JL, Eguiarte LE, Santamaría RL et al. Genomic lineages of *Rhizobium etli* revealed by the extent of nucleotide polymorphisms and low recombination. BMC Evol Biol. 2011;11:305. 10.1186/1471-2148-11-305. 22004448 PMC3215678

[bib2] Adjei JA, Aserse AA, Yli-Halla M et al. Phylogenetically diverse *Bradyrhizobium* genospecies nodulate bambara groundnut (*Vigna subterranea* L. Verdc) and soybean (*Glycine max* L. Merril) in the northern savanna zones of Ghana. FEMS Microbiol Ecol. 2022;98:1–17. 10.1093/femsec/fiac043. PMC9329091 35404419

[bib3] Aguilar OM, López MV, Riccillo PM et al. Prevalence of the *Rhizobium etli*-like allele in genes coding for 16S rRNA among the indigenous rhizobial populations found associated with wild beans from the Southern Andes in Argentina. Appl Environ Microb. 1998;64:3250–524. 10.1128/AEM.64.9.3520-3524.1998. PMC106759 9726909

[bib4] Aguilar OM, Riva O, Peltzer E. Analysis of *Rhizobium etli* and of its symbiosis with wild *Phaseolus vulgaris* supports coevolution in centers of host diversification. P Natl Acad Sci USA. 2004;101:13548–53. 10.1073/pnas.0405321101. PMC518792 15340138

[bib5] Anderson JM, Ingram JSI. Tropical Soil Biology and Fertility. A Handbook of Methods. Wallingford: CAB International, 1993.

[bib6] Anyango B, Wilson KJ, Beynon JL et al. Diversity of rhizobia nodulating *Phaseolus vulgaris* L. in two Kenyan soils with contrasting pHs. Appl Environ Microb. 1995;61:4016–21. 10.1128/aem.61.11.4016-4021.1995. PMC1388601 16535165

[bib8] Aserse AA, Rasänen LA, Assefa F et al. Diversity of sporadic symbionts and nonsymbiotic endophytic bacteria isolated from nodules of woody, shrub, and food legumes in Ethiopia. Appl Microbiol Biotechnol. 2013;97:10117–34. 10.1007/s00253-013-5248-4. 24196581

[bib7] Aserse AA, Rasänen LA, Assefa F et al. Phylogeny and genetic diversity of native rhizobia nodulating common bean (*Phaseolus vulgaris* L.) in Ethiopia. Syst Appl Microbiol. 2012;35:120–31. 10.1016/j.syapm.2011.11.005. 22265597

[bib9] Aserse AA, Woyke T, Kyrpides NC et al. Draft genome sequence of type strain HBR26^T^ and description of *Rhizobium aethiopicu*m sp. nov. Stand Genomic Sci. 2017;12:1–16. 10.1186/s40793-017-0220-z. 28163823 PMC5278577

[bib10] Asfaw B, Aserse AA, Asefa F et al. Genetically diverse lentil- and faba bean-nodulating rhizobia are present in soils across Central and southern Ethiopia. FEMS Microbiol Ecol. 2020;96:fiaa015. 10.1093/femsec/fiaa015. 32020182

[bib11] Broughton WJ, Hernandez G, Blair M et al. Beans (*Phaseolus* spp.)—model food legumes. Plant Soil. 2003;252:55–128. 10.1023/A:1024146710611.

[bib12] Clarke KR, Gorley RN. PRIMER (Plymouth Routines in Multivariate Ecological Research) v5: User Manual/Tutorial. Plymouth: Primer-E Ltd, 2001.

[bib13] Cordeiro AB, Ribeiro RA, Helene LCF et al. *Rhizobium esperanzae* sp. nov., a N_2_-fixing root symbiont of *Phaseolus vulgaris* from Mexican soils. Int J Syst Evol Microbiol. 2017;67:3937–45. 10.1099/ijsem.0.002225. 28895521

[bib63_397_164124] Cottenie A . Soil and plant testing as a basis of fertilizer recommendations. FAO soil bulletin. Rome: Food and Agriculture Organization of the United Nations, 1980.

[bib14] Dall'agnol RF, Ribeiro RA, Delamuta JRM et al. *Rhizobium paranaense* sp. nov., an effective N_2_-fixing symbiont of common bean (*Phaseolus vulgaris* L.) with broad geographical distribution in Brazil. Int J Syst Evol Microbiol. 2014;64:3222–9. 10.1099/ijs.0.064543-0. 24972614

[bib15] de Castro Pires R, dos Reis Junior FB, Zilli JE et al. Soil characteristics determine the rhizobia in association with different species of Mimosa in central Brazil. Plant Soil. 2018;423:411–28. 10.1007/s11104-017-3521-5.

[bib17] FAO . Fertilizer consumption (kilograms per hectare of arable land)| data. 2016. https://data.worldbank.org/indicator/AG.CON.FERT.ZS.

[bib16] FAO . World Reference Base for Soil classification and mapping legend. An international soil classification system for naming the soils and creating legend for soil maps. World Soil Resources Report 106, Rome, Italy, 2014.

[bib18] Gaunt M, Turner S, Rigottier-Gois L et al. Phylogenies of *atpD* and *recA* support the small subunit rRNA-based classification of rhizobia. Int J Syst Evol Microbiol. 2001;51:2037–48. 10.1099/00207713-51-6-2037. 11760945

[bib19] Gunnabo AH, Geurts R, Wolde-Meskel E et al. Phylogeographic distribution of rhizobia nodulating common bean (*Phaseolus vulgaris* L.) in Ethiopia. FEMS Microbio Ecol. 2021;97:fiab046. 10.1093/femsec/fiab046 PMC8016211 33724341

[bib20] Hu Y, Corbett MC, Fay AW et al. Nitrogenase Fe protein: a molybdate/homocitrate insertase. Proc Natl Acad Sci USA. 2006;103:17125–30. 10.1073/pnas.0602651103. 17062756 PMC1859896

[bib21] Jjagwe G, Kibwika P, Mazur R et al. The role of smallholder bean farmers in determining farm gate prices for beans in Uganda. Agric Food Secur. 2022;11:45.

[bib22] Kaizzi KC, Byalebeka J, Semalulua O. Optimizing smallholder returns to fertilizer use: bean, soybean and groundnut. Field Crops Res. 2012;127:109–19. 10.1016/j.fcr.2011.11.010.

[bib23] Kebede E . Contribution, utilization, and improvement of legumes-driven biological nitrogen fixation in agricultural systems. Front Sustain Food Syst. 2021;5:767998. 10.3389/fsufs.2021.767998.

[bib24] Konstantinidis KT, Ramette A, Tiedje JM. Toward a more robust assessment of intraspecies diversity, using fewer genetic markers. Appl Environ Microb. 2006 ;72:7286–93. 10.1128/AEM.01398-06. PMC1636164 16980418

[bib25] Laguerre G, Mavingui P, Allard MR et al. Typing of rhizobia by PCR DNA fingerprinting and PCR-restriction fragment length polymorphism analysis of chromosomal and symbiotic gene regions: application to *Rhizobium leguminosarum* and its different biovars. Appl Environ Microb. 1996;62:2029–36. 10.1128/aem.62.6.2029-2036.1996. PMC167981 8787401

[bib26] MAAIF . Agriculture for Food and Income Security, Agriculture Sector Development Strategy and Investment Plan 2010/11-2014/15. Ministry of Agriculture, Animal Industry & Fisheries (MAAIF), Uganda: MAAIF, Entebbe, 2010.

[bib27] Magurran AE . Measuring Biological Diversity. Oxford: Blackwell Publishing, 2004.

[bib28] Martinez-Romero E . Diversity of *Rhizobium–Phaseolus vulgaris* symbiosis: overview and perspectives. Plant Soil. 2003;252:11–23. 10.1023/A:1024199013926.

[bib29] Mazur R, Nakimbugwe D, Ugen M et al. Enhancing nutritional value and marketability of beans through research and strengthening key value chain stakeholders in Uganda and Rwanda. *In* dry Grain Pulses Collaborative Research Support Program (CRSP). U.S. agency for international development, Washington, DC. 2009. pp.14–23.

[bib30] Mhamdi R, Mrabet M, Laguerre G et al. Colonization of *Phaseolus vulgaris* nodules by *agrobacterium*-like strains. Can J Microbiol. 2005;51:105–11. 10.1139/w04-120. 16091768

[bib31] Miranda-Sánchez F, Rivera J, Vinuesa P. Diversity patterns of *Rhizobiaceae* communities inhabiting soils, root surfaces and nodules reveal a strong selection of rhizobial partners by legumes. Environ Microbiol. 2016;18:2375–91. 10.1111/1462-2920.13061. 26395550

[bib32] Mnasri B, Liu TY, Saidi S et al. *Rhizobium a*zibense sp. nov., a nitrogen fixing bacterium isolated from root nodules of *Phaseolus vulgaris*. Int J Syst Evol Microbiol. 2014;64:1501–6. 10.1099/ijs.0.058651-0. 24478208

[bib33] Mousavi SA, Willems A, Nesme X et al. Revised phylogeny of *Rhizobiaceae:* proposal of the delineation of *pararhizobium* gen. nov., and 13 new species combinations. Syst Appl Microbiol. 2015;38:84–90. 10.1016/j.syapm.2014.12.003. 25595870

[bib34] Muoni T, Barnes AP, Öborn I et al. Farmer perceptions of legumes and their functions in smallholder farming systems in east Africa. Int J Agric Sustain. 2019;17:205–18. 10.1080/14735903.2019.1609166.

[bib35] Musandu AAO, Joshua OO. Response of common bean to *Rhizobium* inoculation and fertilisers. J Food Technol Afr. 2001;6:121–5.

[bib36] Mwenda GM, O'Hara GW, De Meyer SE et al. Genetic diversity and symbiotic effectiveness of *phaseolus vulgaris*-nodulating rhizobia in Kenya. Syst Appl Microbiol. 2018;41: 291–9. 10.1016/j.syapm.2018.02.001. 29571921 PMC6052332

[bib37] Perez-Ramirez NO, Rogel M, Wang E et al. Seeds of *phaseolus vulgaris* bean carry *rhizobium etli*. FEMS Microbiol Ecol. 1998;26:289–96. 10.1016/S0168-6496(98)00043-9.

[bib38] Rahi P, Giram P, Chaudhari D et al. *Rhizobium indicum* sp. nov., isolated from root nodules of pea (*Pisum sativum*) cultivated in the Indian trans-Himalayas. Syst Appl Microbiol. 2020;43:126127. 10.1016/j.syapm.2020.126127. 32847793

[bib39] Ribeiro RA, Martins TB, Ormeno-Orrillo E et al. *Rhizobium ecuadorense* sp. nov., an indigenous N_2_-fixing symbiont of the Ecuadorian common bean (*Phaseolus vulgaris* L.) genetic pool. Int J Syst Evol Microbiol. 2015;65:3162–9. 10.1099/ijsem.0.000392. 26297041

[bib40] Ribeiro RA, Rogel MA, López-López A et al. Reclassification of *rhizobium tropici* type a strains as *rhizobium leucaenae* sp. nov. Int J Syst Evol Microbiol. 2012;62:1179–84. 10.1099/ijs.0.032912-0. 21742822

[bib41] Roche P, Maillet F, Plazanet C et al. The common *nodABC* genes of *rhizobium meliloti* are host-range determinants. Proc Natl Acad Sci USA. 1996;93:15305–10. 10.1073/pnas.93.26.15305. 8986807 PMC26400

[bib42] Rodino PA, Santalla M, De Ron AM et al. Co-evolution and migration of bean and rhizobia in Europe. In: Lichtfouse E (ed.), Sociology, Organic Farming, Climate Change and Soil Science. New York: Springer Science+Business Media B.V, 2010, 474.

[bib43] Rogel MA, Ormeno-Orrillo E, Martinez Romero E et al. Symbiovars in rhizobia reflect bacterial adaptation to legumes. Syst Appl Microbiol. 2011;34:96–104. 10.1016/j.syapm.2010.11.015. 21306854

[bib44] Román-Ponce B, Zhang YJ, Vásquez-Murrieta MS. *Rhizobium acidisoli* sp. nov., isolated from root nodules of *phaseolus vulgaris* in acid soils. Int J Syst Evol Microbiol. 2016;66:398–406. 10.1099/ijsem.0.000732. 26530784

[bib45] Rouhrazi K, Khodakaramian G, Velázquez E. Phylogenetic diversity of rhizobial species and symbiovars nodulating *phaseolus vulgaris* in Iran. FEMS Microbiol Lett. 2016;363:1–8. 10.1093/femsle/fnw024. 26832644

[bib46] Rurangwa E, Vanlauwe B, Giller KE. Benefits of inoculation, P fertilizer and manure on yields of common bean and soybean also increase yield of subsequent maize. Agric Ecosyst Environ. 2018; 261:219–29. 10.1016/j.agee.2017.08.015. 29970950 PMC5946694

[bib47] Sebuwufu G, Mazur R, Ugen M et al. Using improved varieties and fertility enhancements for increasing yield of common beans (*Phaseolus vulgaris* L.) grown by small-landholder farmers in Uganda. Afr J Agric Res. 2015;10:4795–805.

[bib48] Sibiko K, Ingasia O, Gido E et al. Analysis of the determinants of productivity and technical efficiency among smallholder common bean farmers in Eastern Uganda. JEDS. 2013;4:44–55. 10.19026/crjet.5.5524.

[bib49] Simiyu NSW, Tarus D, Watiti J et al. Efective regulation of bio fertilizers and biopesticides: a potential avenue to increase agricultural productivity. IITA; 2013. COMPRO II policy series No. 1:1–3.

[bib50] Somasegaran P, Hoben H. Handbook for Rhizobia: methods in Legume-Rhizobium Technology. Berlin: Springer Science and Business Media, 2012.

[bib51] Souza V, Bain J, Silva C et al. Ethnomicrobiology: do agricultural practices modify the population structure of the nitrogen fixing bacteria *rhizobium etli* biovar phaseoli?. J Ethnobiol. 1997;17:249–66.

[bib52] Staden R, Beal KF, Bonfield JK. The staden package, 1998. In: Bioinformatics Methods and Protocols. Humana Press, Totowa, NJ: Springer, 2000, 115–30. 10.1385/1-59259-192-2:115 10547834

[bib53] Stamatakis A . RAxML version 8: a tool for phylogenetic analysis and post-analysis of large phylogenies. Bioinformatics. 2014;30:1312–3. 10.1093/bioinformatics/btu033. 24451623 PMC3998144

[bib54] Tamura K, Stecher G, Kumar S. MEGA11: molecular Evolutionary Genetics Analysis Version 11. Mol Biol Evol. 2021;38:3022–7. 10.1093/molbev/msab120. 33892491 PMC8233496

[bib55] Tong W, Li X, Huo Y et al. Genomic insight into the taxonomy of *rhizobium* genospecies that nodulate *Phaseolus vulgaris*. Syst Appl Microbiol. 2018;41:300–10. 10.1016/j.syapm.2018.03.001. 29576402

[bib56] UBOS (Uganda Bureau of Statistics) . Uganda Census of Agriculture 2008/2009. Volume IV: Crop Area and Production Report. Uganda, Kampala, 2010.

[bib57] Van Reeuwijk LP . Technical paper 9. Procedures for soil analysis. In 6th edn. Int Soil Ref Inf Cent Food Agric Organ United Nation. Published online. 2002.

[bib58] Wekesa C, Kiprotich K, Okoth P. Molecular characterization of indigenous rhizobia from Kenyan soils nodulating with common beans. Int J Mol Sci. 2023;24:9509. 10.3390/ijms24119509. 37298462 PMC10253646

[bib60] Yan J, Yan H, Liu LX. *Rhizobium hidalgonense* sp. nov., a nodule endophytic bacterium of *phaseolus vulgaris* in acid soil. Arch Microbiol. 2017;199:97–104. 10.1007/s00203-016-1281-x. 27557842

[bib61] Zhang B, Du N, Li Y et al. Distinct biogeographic patterns of rhizobia and non-rhizobial endophytes associated with soybean nodules across China. Sci Total Environ. 2018;643:569–78. 10.1016/j.scitotenv.2018.06.240. 29945091

[bib62] Zhang YM, Li Y, Chen WF et al. Biodiversity and biogeography of rhizobia associated with soybean plants grown in the north China plain. Appl Environ Microb. 2011;77:6331–42. 10.1128/AEM.00542-11. PMC3187167 21784912

