# Supplementary material for: Phylogenetic diversity of Rhizobium species recovered from nodules of common beans (Phaseolus vulgaris L.) in fields in Uganda: R. phaseoli, R. etli, and R. hidalgonense
Source: FEMS Microbiol Ecol. 2024 Sep 12;100(11):fiae120. doi: 10.1093/femsec/fiae120 (PMC11556343; doi:10.1093/femsec/fiae120)
Supplement: fiae120_Supplemental_Files [file fiae120_supplemental_files.zip › Supplemntary_data_figures_revised.docx]

**LIR_A**

**LUW_J**

**LUW_A**

*R. etli* HBR2 (JN580720)

*A. pusense* FDAARGOS633 (CP050898)

*A. pusense* CFBP5875 (CP039894)

**TOR_G**

**IBA_A**

*A. tumefaciens* CFBP7129 (CP039922)

*A. leguminum* AT13 (CP119971)

*A. tumefaciens* SJ003 (CP048537)

**ISI_F**

*A. fabrum* C58^T^ (AE007869)

*R. leucaenae* CFN299^T^ (NZLNCJ01000015)

*R. hainanense* CCBAU57015^T^ (NZFMAC01000028)

*R. tropici* CIAT899^T^ (CP004015)

*R. lusitanum* P1-7^T^ (NZFMAF01000054)

*B. elkanii* USDA76^T^ (CP066356)

*B. japonicum* USDA6^T^ (NZAXAV01000011)

90

99

100

95

58

99

91

70

96

100

75

99

100

99

65

72

100

99

100

99

100

97

75

100

66

92

100

88

100

73

59

100

97

100

67

74

65

98

83

66

**0.20**

*R. hidalgonense* CB782 (CP007067)

**IBA_C**

**MUK_I**

**KLE_F**

*R. hidalgonense* FH14^T^ (NZ_LODW01000085.1), JKLM19E (CP054027)

**FOP_I**

*Rhizobium* sp. HBR42 (JN580741)

**FOP_D**

**KLE_A**

**ISI_H**

**KLE_E**

**I.** *Rhizobium hidalgonense*

*R. acidisoli* FH23^T^ (CP034998)

**ISI_B**

**KLE_H**

**KLE_B**

*R. ecuadorense* CNPSO671^T^ (NZLFIO01001107)

**II**. *Rhizobium ecuadorense*

*Rhizobium* sp. N941 (CP013643)

*R. indigoferae* CCBAU71042^T^ (JN580755)

*R. leguminosarum* ATCC10004^T^ (JAWXXW010000003)

*R. sophorae* CCBAU03386^T^ (NZJABFCN010000024)

*R. laguerreae* FB206^T^ (NZMRDM01000033)

*R. fabae* CCBAU33202^T^ (NZML133658)

*R. pisi* DSM30132^T^ (RJJT01000044)

*Rhizobium* sp. CIAT894 (CP020947)

*R. phaseoli* R723 (CP013527)

*R. etli* IE4803 (CP007641)

*R. sophoriradicis* CC511 (CP104152)

*Rhizobium* sp. Kim5 (CP021124)

*R. sophoriradicis* CCBAU03470^T^(NZRQIH01000006)

**ISI_D**

*R. phaseoli* S3 (CP064931)

*R. phaseoli* S2 (NZJAAVVN010000047)

*R. phaseoli* HBR53 (JN580744)

*R. phaseoli* R611 (CP013547)

**LUW_E**

*R. phaseoli* N671 (CP013574)

*R. phaseoli* R620 (CP013542)

**MAY_G**

*R. phaseoli* B3 (NZJAAVVP010000008)

**LUW_G**

**MUK_A**

*R. phaseoli* N771 (CP013568)

**MUK_G**

**TOR_B**

*R. phaseoli* R744 (CP013522)

**NAM_J**

**NAM**_J

*R. phaseoli* HBR10 (JN580726.)

*R. phaseoli* CIAT652 (CP001074)

*R. phaseoli* N831 (CP013563)

*R. phaseoli* ATCC14482^T^ (NZML133574)

*R. phaseoli* Brasil5 (CP020896)

**MAY_I**

*R. phaseoli* R630 (CP013537)

**LIR_B**

**LIR_D**

**IV.** *Rhizobium phaseoli*

*R. aethiopicum* HBR23 (JN580737)

*R. aethiopicum* HBR26^T^ (NZFMAJ01000038)

*R. etli* CFN42^T^ (CP000133)

*R. etli* NXC12 (CP020906)

*Rhizobium* sp. NXC14 (CP021030)

*R. etli* HBR5(JN580723)

**LIR_C**

**MAY_J**

**MAY_A**

**IBA_I**

**IBA_E**

**NAM_I**

*Rhizobium* sp. N741 (CP013595)

*Rhizobium* sp. N113 (CP013517)

*Rhizobium* sp. N1341 (CP013505)

*Rhizobium* sp. N871 (CP013590)

*R. etli* HBR4 (JN580722)

**III.** *Rhizobium sophoriradicis*

**NAM_C**

**V.** *Rhizobium etli*

**VI.** *Agrobacterium pusense*

**VIII.** *Agrobacterium tumefaciens*

**IBA_D**

**VIII.** *Agrobacterium* species

**Figure S1.** Displays a Maximum Likelihood (RAxML) phylogeny based on the *rpoB* gene, showing the taxonomic relationships between bacterial strains recovered from nodules of common beans in Ugandan (**bold**) and closely related species of *Rhizobium–Agrobacterium*. Bootstrap values ≥50% are shown at branch nodes. The scale bar (0.2) represents estimated nucleotide substitution rates. s. Gene sequence accession numbers of the references are in parenthesis, with type strains marked with superscript 'T'. *B. japonicum* USDA 6^T^ and USDA 76^T^ sequences are included as outgroup references. *B, Bradyrhizobium; R, Rhizobium; A, Agrobacterium*.

*R. leucaenae* CFN299^T^ (NZLNCJ01000024)

*R. hainanense* CCBAU57015^T^ (NZFMAC01000017)

*R. tropici* CIAT899^T^ (CP004015)

*R. lusitanum* P1-7T (NZFMAF01000024)

*A. tumefaciens* ATCC4720^T^ (JAAQPP010000003)

*A. fabrum* C58^T^ (AE007869)

*A. pusense* CFBP5875 (CP039894)

*A. pusense* FDAARGOS633 (CP050898)

*A. tumefaciens* CFBP7129 (CP039922)

*A. leguminum* AT13 (CP119971)

*A. tumefaciens* SJ003 (CP048537)

*B. japonicum* USDA6^T^ (NZAXAV01000007)

*B. elkanii* USDA76^T^ (CP066356)

91

99

100

100

89

82

100

69

55

63

76

96

83

98

98

99

58

94

64

54

50

99

100

88

100

100

100

78

100

52

54

65

90

100

98

58

100

56

87

**0.10**

*R. hidalgonense* FH14^T^(LODW01000041), CB782 (CP007067)

**KL_F**

*R. hidalgonense* CB782 (CP007067)

**IBA_C**

**FOP_I**

**KLE_A**

**KLE_E**

**ISI_H**

**FOP_D**

**I***. Rhizobium hidalgonense*

*R. acidisoli* FH23^T^ (CP034998)

*Rhizobium* sp. CIAT894 (CP020947)

*Rhizobium* sp. N941 CP013643

*Rhizobium* sp. N324 (CP013630)

**KLE_H**

**ISI_B**

**KLE_B**

*R. ecuadorense* CNPSO671^T^ (NZLFIO01000207)

**II**. *Rhizobium ecuadorense*

*R. indigoferae* CCBAU71042^T^ (NZJABFCO010000047)

*R. leguminosarum* ATCC10004^T^ (JAWXXW010000003)

*R.sophorae* CCBAU03386^T^ (NZJABFCN010000019)

*R. laguerreae* FB206^T^ (NZMRDM01000010)

*R. sophoriradicis* CC511 (CP104152)

*R. etli* IE4803 (CP007641)

*R. sophoriradicis* CCBAU03470^T^ (RQIH01000017)

*Rhizobium* sp. Kim5 CP021124

**ISI_D**

*R. phaseoli* ATCC14482^T^(RJJV01000002)

*R. phaseoli* R630 (CP013537)

**LIR_B**

**LIR_D**

*R. phaseoli* R611 (CP013547)

*R. phaseoli* N671 (CP013574)

**TOR_B**

*R. phaseoli* N771 (CP013568)

**LUW_G**

**MAY_G**

*R. phaseoli* R620 (CP013542)

**MUK_A**

*R. phaseoli* HBR53

*R. phaseoli* HBR10

**MAY_I**

*R. phaseoli* N831 CP013563

**LUW_E**

**NAM_J**

**MUK_G**

*R. phaseoli* R744 (CP013522)

*R. etli* CIAT652 (CP001074)

*R. phaseoli* Brasil5 (CP020896)

**IV.** *Rhizobium phaseoli*

*R. etli* NXC12 (CP020906)

*R. etli* CFN42^T^ (CP000133)

**LUW_A**

**LIR_A**

**LUW_J**

**NAM_C**

*Rhizobium* sp. N113 (CP013517)

Rhizobium sp. N1341 (CP013505)

Rhizobium sp. N741 (CP013595)

*Rhizobium* sp. N871 (CP013590)

**IBA_I**

**IBA_E**

**MAY_A**

**MAY_J**

**LIR_C**

**NAM_I**

*R- etli* HBR5

*Rhizobium* sp. NXC14 (CP021030)

**V.** *Rhizobium etli*

*R. fabae* CCBAU33202^T^ (RJJU01000012)

*R. pisi* DSM30132^T^ (RJJT01000013)

*R. aethiopicum* HBR26T FMAJ01000003

**III.** *Rhizobium sophoriradicis*

**TOR_G**

**ISI_F**

**IBA_A**

**VII.** *Agrobacterium tumefaciens*

**IBA_D**

**VI.** *Agrobacterium pusense*

**VIII.** *Agrobacterium* species

**Figure S2.** Displays a Maximum Likelihood (RAxML) phylogeny based on the *dnaK* gene, showing the taxonomic relationships between bacterial strains recovered from nodules of common beans in Ugandan (**bold**) and closely related species of *Rhizobium–Agrobacterium*. Bootstrap values ≥50% are shown at branch nodes. The scale bar (0.1) represents estimated nucleotide substitution rates. Gene sequence accession numbers of the references are in parenthesis, with type strains marked with superscript 'T'. *B. japonicum* USDA 6^T^ and USDA 76^T^ sequences are included as outgroup references. *B, Bradyrhizobium;* *R, Rhizobium; A, Agrobacterium.*

*R. aethiopicum* HBR23 (JN580701)

*R. aethiopicum* HBR26^T^ (JN580703)

*R. hidalgonense* CB782 (CP007067)

**IBA_C**

**MUK_I**

**KLE_F**

**ISI_H**

**KLE_E**

*R. hidalgonense* FH14^T^ (KJ921081), JKLM19E (CP054027)

**FOP_I**

*Rhizobium* sp. HBR42 (JN580705)

**I.** *Rhizobium hidalgonense*

*R. ecuadorense* CNPSO671^T^ (NZLFIO01001796)

**ISI_B**

**KLE_B**

**KLE_H**

**II**. *Rhizobium ecuadorense*

*Rhizobium* sp. CIAT894 (CP020947)

*R. indigoferae* CCBAU71042^T^ (JABFCO010000007)

*R. leguminosarum* USDA2370^T^ (MRDL01000036)

*R. fabae* CCBAU33202^T^ (ML133645)

*R. pisi* DSM30132^T^ (RJJT01000016)

*R. acidisoli* FH23^T^ (CP034998)

*Rhizobium* sp. N941 (CP013643)

*Rhizobium* sp. N324 (CP013630)

*R. laguerreae* FB206^T^ (MRDM01000014)

**MAY_A**

**MAY_J**

**IBA_I**

**IBA_E**

**NAM_I**

**LIR_C**

*Rhizobium* sp. NXC14 (CP021030)

*R. etli* HBR5

*R. etli* CFN42^T^ (CP000133)

*R. etli* NXC12 (CP020906)

*Rhizobium* sp. N871 (CP013590)

*Rhizobium* sp. N741 (CP013595)

*Rhizobium* sp. N113 (CP013517)

*Rhizobium* sp. N1341 (CP013505)

**LUW-A**

**LUW-J**

*R. etli* HBR2 (JN580685)

**LIR_A**

**NAM_C**

**V.** *Rhizobium etli*

*Rhizobium* sp. Kim5 (CP021124)

**ISI_D**

*R. sophoriradicis* CC511 (CP104152)

*R. etli* IE4803 (CP007641)

**NAM_J**

**MUK_G**

**LUW_E**

*R. phaseoli* ATCC14482^T^ (RJJV01000010)

*R. phaseoli* Brasil5 (CP020896)

*R. phaseoli* R630 (CP013537)

*R. phaseoli* N831 (CP013563)

*R. phaseoli* R744 (CP013522)

**LIR_D**

**LIR_B**

**MAY_I**

*R. phaseoli* R723 (CP013527)

**TOR_B**

*R. phaseoli* CIAT652 (CP001074)

*R. phaseoli* R620 (CP013542)

*R. phaseoli* R611 (CP013547)

*R. phaseoli* N771 (CP013568)

*R. phaseoli* N671 (CP013574)

*R. phaseoli* HBR10

*R. phaseoli* HBR53

*R. phaseoli* S3 (CP064931)

**LUW_G**

**MUK_A**

**MAY_G**

**IV.** *Rhizobium phaseoli*

*R. lusitanum* P1-7^T^ (EF639841)

*R. tropici* CIAT899^T^ (CP004015)

*R. tropici* CFN299^T^ (AF169583)

*A. fabrum* C58^T^ (AE007869)

*A. pusense* FDAARGOS633 (CP050898)

*A. leguminum* AT13 (CP119971)

*A. tumefaciens* CFBP7129 (CP039922)

*A. tumefaciens* SJ003 (CP048537)

*B. elkanii* USDA76^T^ (CP066356)

*B. japonicum* USDA6^T^ (AP012206)

99

69

79

95

58

99

99

83

83

99

84

85

71

99

96

95

99

99

62

79

98

99

99

66

99

99

67

99

99

84

99

67

82

99

90

98

75

90

83

52

99

99

**0.050**

**III.** *Rhizobium sophoriradicis*

**TOR_G**

**IBA_D**

**VII.** *Agrobacterium* species

**VI.** *Agrobacterium pusense*

**ISI_F**

**IBA_A**

**VIII.** *Agrobacterium tumefaciens*

**Figure S3.** Displays a Maximum Likelihood (RAxML) phylogeny based on the *glnII* gene, showing the taxonomic relationships between bacterial strains recovered from nodules of common beans in Ugandan (**bold**) and closely related species of *Rhizobium–Agrobacterium*. Bootstrap values ≥50% are shown at branch nodes. The scale bar (0.05) represents estimated nucleotide substitution rates. Gene sequence accession numbers of the references are in parenthesis, with type strains marked with superscript 'T'. *B. japonicum* USDA 6^T^ and USDA 76^T^ sequences are included as outgroup references. *B, Bradyrhizobium; R, Rhizobium; A, Agrobacterium*.

*R. aethiopicum* HBR26^T^ (NZFMAJ01000018)

*R. leucaenae* CFN299^T^ (NZLNCJ01000016)

*R. hainanense* CCBAU57015^T^ (HQ438236)

*R. tropici* CIAT899^T^ (CP004015)

*R. lusitanum* P1-7^T^ (NZFMAF01000002)

*A. fabrum* C58^T^ (AE007869)

*A.tumefaciens* ATCC4720^T^ (FR695218)

*A.pusense* CFBP5875 (CP039894)

*A.pusense* FDAARGOS633 (CP050898)

*A. tumefaciens* SJ003 (CP048537)

*A. tumefaciens* CFBP7129 (CP039922)

*B. elkanii* USDA76^T^ (CP066356)

*B. japonicum* USDA6^T^ (AB070586)

88

62

80

99

88

96

68

71

87

100

95

87

81

99

94

92

52

100

96

97

100

63

84

100

87

81

83

96

100

88

79

96

94

68

100

82

58

66

100

98

60

51

100

64

100

65

**0.20**

*R.hidalgonense* FH14^T^(NZ NWSY01000030)

**MAY_J**

IBA_D

**IBA_A**

**ISI_F**

**VI.** *Agrobacterium pusense*

**FOP_D**

**KLE_E**

**KLE_A**

**ISI_H**

*R. hidalgonense* CB782 (CP007067)

**FOP_I**

*R. hidalgonense* JKLM19E (CP054027)

**KLE_F**

**MUK_I**

**IBA_C**

**I.** *Rhizobium hidalgonense*

**ISI_B**

**KLE_H**

**KLE_B**

*R. ecuadorense* CNPSO671^T^ (NZLFIO01000871)

*Rhizobium* sp. N941 (CP013643)

**II.** *Rhizobium ecuadorense*

*R. acidisoli* FH23^T^ (CP034998)

*R. pisi* DSM30132^T^ (NZRJJT01000027)

*R. fabae* CCBAU33202^T^ (NZML133661)

*R. laguerreae* FB206^T^ (NZMRDM01000011)

*R. sophorae* CCBAU03386^T^ (NZJABFCN010000066)

*R. leguminosarum* USDA2370^T^ (QBLB01000011)

*R. indigoferae* CCBAU71042^T^ (NZJABFCO010000023)

*Rhizobium* sp. CIAT894 (CP020947)

*Rhizobium* sp. N741 (CP013595)

*Rhizobium* sp. N1341 (CP013505)

Rhizobium sp. N113 (CP013517)

*Rhizobium* sp. N871 (CP013590)

**LUW_J**

**NAM_C**

**LUW_A**

**LIR_A**

*R. etli* IE4803 (CP007641)

*Rhizobium* sp. Kim5 (CP021124)

*R. sophoriradicis* CC511 (CP104152) R. sophoriradicis

*R. sophoriradicis* CCBAU03470^T^ (NZRQIH01000026)

*R. phaseoli* ATCC14482^T^ (NZML133567)

*R. phaseoli* Brasil5 (CP020896)

**MAY_I**

*R. phaseoli* R744 (CP013522)

*R. phaseoli* CIAT652 (CP001074)

*R. phaseoli* R620 (CP013542)

*R. phaseoli* N671 (CP013574)

*R. phaseoli* HBR10

**LUW_G**

**MAY_G**

*R. phaseoli* S3 (CP064931)

*R. phaseoli* R611 (CP013547)

*R. phaseoli* HBR53

*R. phaseoli* N771 (CP013568)

**MUK_A**

**TOR_B**

*R. phaseoli* N831 (CP013563)

*R. phaseoli* R630 (CP013537)

**LIR_D**

*R. phaseoli* R723 (CP013527)

**LIR_B**

**MUK_G**

**LUW_E**

**NAM_J**

**IV.** *Rhizobium phaseoli*

*R. etli* CFN42^T^ C(P000133)

*R. etli* NXC12 (CP020906)

*Rhizobium* sp. NXC14 (CP021030)

*R. etli* HBR5

**NAM_I**

**MAY_A**

**IBA_I**

**LIR_C**

**V.** *Rhizobium etli*

**TOR_G**

**VIII.** *Agrobacterium tumefaciens*

**VII.** *Agrobacterium species*

**IBA_E**

**III.** *Rhizobium* *sophoriradicis*

**ISI_D**

**Figure S4.** Displays a Maximum Likelihood (RAxML) phylogeny based on the *gyrB* gene, showing the taxonomic relationships between bacterial strains recovered from nodules of common beans in Ugandan (**bold**) and closely related species of *Rhizobium–Agrobacterium*. Bootstrap values ≥50% are shown at branch nodes. The scale bar (0.2) represents estimated nucleotide substitution rates. Gene sequence accession numbers of the references are in parenthesis, with type strains marked with superscript 'T'. *B. japonicum* USDA 6^T^ and USDA 76^T^ sequences are included as outgroup references. *B, Bradyrhizobium; R, Rhizobium; A, Agrobacterium*.

100

69

85

68

66

100

50

100

100

100

86

91

98

80

92

73

65

96

76

65

51

99

100

90

52

56

99

100

**0.10**

R. etli CFN42^T^ (CP000133)

*Rhizobium* sp. N113 (CP013517)

*Rhizobium* sp. N741 (CP013595)

*Rhizobium* sp. N871 (CP013590)

*R. etli* HBR5

**LIR_C**

**MAY_A**

**IBA_E**

**NAM_I**

**MAY_J**

**IBA_I**

**V.** *Rhizobium etli*

*Rhizobium* sp. CIAT894 (CP020947)

*Rhizobium* sp. NXC14 (CP021030)

*R. tropici* CIAT899^T^ (CP004015)

*R. hainanense* CCBAU 57015^T^ (NZ FMAC01000004)

*R. lusitanum* P1-7^T^ (NZFMAF01000002)

*R. leucaenae* CFN299^T^ (NZLNCJ01000004)

*A. pusense* FDAARGOS633 (CP050898)

**TOR_G**

*A. pusense* CFBP5875 (CP039894)

**VI.** *Agrobacterium pusense*

*A. leguminum* AT13 (CP119971)

**ISI_F**

*A. tumefaciens* CFBP7129 (CP039922)

**IBA_A**

**VIII.** *Agrobacterium tumefaciens*

*A. fabrum* C58^T^ (AE007869)

**IBA_D**

*A. tumefaciens* SJ003 (CP048537)

*B. elkanii* USDA76^T^ (CP066356)

*B. japonicum* USDA6^T^ (NZAXAV01000007)

69

95

96

91

96

98

100

90

73

100

100

100

100

72

78

85

100

100

74

89

79

**MUK_I**

**KLE_F**

**FOP_I**

*R. hidalgonense* FH14*^T^*(NZ_LODW01000082.1), CB782 (CP007067)

**IBA_**C

*R. hidalgonense* JKLM19E (CP054027)

*Rhizobium* sp. NAK334 (MG288764)

**KLE**_E

**KLE_A**

**ISI_H**

**FOP_D**

*Rhizobium* sp. NAK382 (MG288772)

*Rhizobium* sp. NAK295 (MG288757)

*Rhizobium* sp. NAK214 (MG288744)

*Rhizobium* sp. NAK227 (MG288746)

**I.** *Rhizobium hidalgonense*

**KLE_H**

**KLE_B**

**ISI_B**

*R. ecuadorense* CNPSO671^T^ (NZLFIO01001065)

**II.** *Rhizobium ecuadorense*

*R. laguerreae* FB206^T^ (NZMRDM01000011)

*R. sophorae* CCBAU03386^T^ (NZJABFCN010000022)

*R. acidisoli* FH23^T^ (CP034998)

*R. indigoferae* CCBAU71042^T^ (NZJABFCO010000023)

*R. leguminosarum* ATCC10004^T^ (JAWXXW010000003)

*Rhizobium* sp. N324 (CP013630)

*Rhizobium* sp. N941 (CP013643)

*R. faba*e CCBAU33202^T^ (NZML133652)

*R. pisi* DSM30132^T^ (NZRJJT01000010)

*Rhizobium* sp. IE4771 (CP006986)

*R. etli* IE4803 (CP007641)

*R. sophoriradicis* CC511 (CP104152)

Rhizobium sp. NAK387 (MG288773)

*Rhizobium* sp. NAK368 (MG288770)

*Rhizobium* sp. NAK378 (MG288771)

*Rhizobium* sp. Kim5 (CP021124)

**ISI_D**

*R. sophoriradicis* CCBAU03470^T^ (NZRQIH01000005)

*R. aethiopicum* HBR26^T^ (NZFMAJ01000010)

**LUW_A**

**LUW_J**

**LIR_A**

**NAM_C**

*Rhizobium* sp. NAK245 (MG288750)

*R. phaseoli* N771 (CP013568)

*R. phaseoli* N671 (CP013574)

*R. phaseoli* R611 (CP013547)

*R. phaseoli* R620 (CP013542)

*R. phaseoli* HBR53

*R. phaseoli* B3 (NZJAAVVP010000018)

*R. phaseoli* S2 (NZJAAVVN010000013)

*R. phaseoli* S3 (CP064931)

*R. phaseoli* HBR10

**MUK_A**

**LUW_G**

**TOR_B**

*Rhizobium* sp. NAK242 (MG288749)

*Rhizobium* sp. NAK103 (MG288740)

*Rhizobium* sp. NAK407 (MG288774)

*Rhizobium* sp. NAK231 (MG288747)

**NAM_J**

**LUW_E**

**MUK_G**

**MAY_G**

*R. phaseoli* N831 (CP013563)

*R. phaseoli* R744 (CP013522)

*R. phaseoli* CIAT652 (CP001074)

*R. phaseoli* R630 (CP013537)

**LIR_B**

**LIR_D**

*R. phaseoli* R723 (CP013527)

*R. phaseoli* Brasil5 (CP020896)

**MAY_I**

*R. phaseoli* ATCC14482^T^ (ML133567)

**IV.** *Rhizobium phaseoli*

*R. etli* NXC12 (CP020906)

**III.** *Rhizobium sophoriradicis*

**VII.** *Agrobacterium* species

**Figure S5.** Displays a Maximum Likelihood (RAxML) phylogeny based on the atpD gene, showing the taxonomic relationships between bacterial strains recovered from nodules of common beans in Ugandan (**bold**) and closely related species of *Rhizobium–Agrobacterium*. Bootstrap values ≥50% are shown at branch nodes. The scale bar (0.2) represents estimated nucleotide substitution rates. Gene sequence accession numbers of the references are in parenthesis, with type strains marked with superscript 'T'. *B. japonicum* USDA 6^T^ and USDA 76^T^ sequences are included as outgroup references. *B, Bradyrhizobium; R, Rhizobium; A, Agrobacterium*.
