# Supplementary material for: Phylogenetic diversity of Rhizobium species recovered from nodules of common beans (Phaseolus vulgaris L.) in fields in Uganda: R. phaseoli, R. etli, and R. hidalgonense
Source: FEMS Microbiol Ecol. 2024 Sep 12;100(11):fiae120. doi: 10.1093/femsec/fiae120 (PMC11556343; doi:10.1093/femsec/fiae120)
Supplement: fiae120_Supplemental_Files [file fiae120_supplemental_files.zip › supplemntary_data_tables_revised.doc]

Table S1_ supplementary data. Distribution of isolates in sampling sites.

| **Site** | **Agro-ecological zone** | **Zone characteristics** | **Latitude** | **Longitude** | **Soil texture** | **Soil colour** | **Strain** |
| --- | --- | --- | --- | --- | --- | --- | --- |
| Lira | Northern moist farmland  (Northern System)) | Bi-modal annual rainfall (1000-2000 mm) adequate for most crops but the intensity of the dry season requires that drought tolerant annuals are cultivated (finger millet, sesame, cassava and sorghum). Tobacco and cotton are major cash crops. There is short grassland where communal grazing abounds. | 2.25810 | 32.8874 | Clay-loam | brown | LIR_D, LIR_B, LIR_A, LIR_J, LIR_I, LIR_G, LIR_F, LIR_E, LIR_C |
| Namutumba | Lake Victoria crescent  (Banana-Robusta Coffee System) | Evenly distributed bi-modal rainfall on medium to high productivity soils. Vegetation is mainly forest/savanna mosaic with pastures suitable for intensive livestock management. Banana and coffee are the main crops with root crops on the increase. Livestock is generally not integrated into the system but it can be an important source of income. | 0.84930 | 33.6623 | fine-sand | Red-brown | NAM_J, NAM_D, NAM_C, NAM_I, NAM_H, NAM_E, NAM_B, NAM_F |
| Mayuge |  |  | 0.21830 | 33.5728 | Sandy- clay-loam | Yellow-red | MAY_I, MAY_E, MAY_G, MAY_H, MAY_F, MAY_D, MAY_C, MAY_B, MAY_J, MAY_A |
| Luwero |  |  | 0.82710 | 32.6277 | Silt-loam | Dark-black- gray | LUW_F, LUW_E, LUW_H, LUW_G, LUW_F2, MUK_H  LUW_I, LUW_B, LUW_J  LUW_D, LUW_C, LUW_A |
| Mukono |  |  | 0.28350 | 32.7633 | Clay-loam | brown | MUK_I, MUK_E, MUK_C, MUK_A, MUK_J, MUK_G  MUK_F |
| Kabale | Southwestern highlands  (montane system/kigezi afro montane) | High land altitude (> 2000m above sea level) with high bi-modal rainfall >1000 mm/pa. High population density with smaller sized holdings. Dominantly potatoes and legumes (beans and field peas). | 1.25235 | 29.9872 | Silt-loam | Yellow- brown | KLE_G, KLE_F, KLE_I, KLE_E  KLE_A, KLE_B, KLE_H, KLE_D |
| Isingiro | Southwestern farmlands  (western banana coffee cattle system) | There is greater reliance on annual food crops (millet, sorghum and maize) since rainfall is less stable than under the Banana-Coffee System. In the drier areas livestock is a main activity.  It is one of the leading bean production areas in the country | 0.84350 | 30.8039 | Sand-loam | Brown | ISI_H, ISI_B, ISI_D, ISI_A, ISI_C, ISI_G, ISI_F |
| Ibanda |  |  | 0.09650 | 30.5740 | Very fine sand | Dark-yellow- brown | IBA_D, IBA_C, IBA_I, IBA_E  IBA_F, IBA_B, IBA_A |
| Fort portal | Western medium-highland farmlands  (West Nile System) | Rainfall patterns are similar to the northern system with greater rain at higher elevation. Intercropping is common with a wide variety of crops. The system is in the sub-humid zone and livestock activities are limited by the presence of tsetse fly. | 0.58510 | 30.2513 | Sandy- clay-loam | Black | FOP_I, FOP_E, FOP_C, FOP_D |
| Tororo | Mount Elgon Farmlands  (Teso system) | Characterised by bi-modal rain falling on sandy-loam medium to low fertility soils. Main staples are cassava, millet and maize. There is short grassland ideal for grazing. Use of crop residues is very common. | 0.68710 | 34.0641 | Sand-loam | Dark-yellow- brown | TOR_B, TOR_G, TOR_H |

The soil types were classified using the GPS coordinates information of the sites and the World Reference Base classification system (IUSS Working Group WRB., 2014).

Agro-ecological zone charcterstices (MAAIF 2010). Agriculture for Food and Income Security, Agriculture Sector Development Strategy and Investment Plan 2010/11-2014/15, Ministry of Agriculture, Animal Industry & Fisheries (MAAIF).

Table S2_ supplementary data. Information of nucleotide sequence used in the phylogenetic analysis.

|  | **Number of taxa included** | ***Number of alignment sites (bp)** | **Conserved sites (bp)** | **Variable sites (bp)** | **Parsimony-informative sites (bp)** | **Singleton**  **(bp)** |
| --- | --- | --- | --- | --- | --- | --- |
| *recA* | 148 | 464 | 281 | 183 | 170 | 13 |
| *rpoB* | 95 | 945 | 570 | 375 | 329 | 46 |
| *dnaK* | 88 | 1013 | 563 | 450 | 306 | 144 |
| *glnII* | 88 | 863 | 556 | 307 | 250 | 57 |
| *gyrB* | 88 | 671 | 327 | 344 | 306 | 38 |
| *atpD* | 104 | 460 | 308 | 152 | 142 | 10 |
| *recA-rpoB-dnaK-glnII-gyrB-atpD* | 78 | 4416 | 2624 | 1792 | 1471 | 321 |
| *nodC* | 56 | 1020 | 521 | 519 | 452 | 67 |
| *nifH* | 63 | 651 | 397 | 254 | 148 | 106 |
|  |  |  |  |  |  |  |

* Sequence sites of the alignment in base pair used for tree construction.

Table S3_ supplementary data. Distribution and diversity of the species identified across the sampling sites as determined with diversity indices.

| **Site** | ***R. phaseoli*** | ***R. etli*** | ***R. hidalgonense*** | ***Agrobacterium* spp.** | ***R. ecuadorense*** | ***Rhizo_sp.CIAT894*** | ***R. sophorae*** | **Total Number of strains site** | **Number of species identified per site** | **Species richness Margalef's index(D)** | **Shannon-Wiener Index (H')** | **Simpson's Index (λ)** | **Pilou evenness (J)** | **Simpson's Index (DS)** | **TRUE_SHANNON** |
| --- | --- | --- | --- | --- | --- | --- | --- | --- | --- | --- | --- | --- | --- | --- | --- |
| Lira | 2 | 7 | 0 | 0 | 0 | 0 | 0 | **9** | **2** | 0.50 | 0.53 | 0.35 | 0.50 | 0.71 | 1.70 |
| Namutumba | 1 | 7 | 0 | 0 | 0 | 0 | 0 | **8** | **2** | 0.48 | 0.40 | 0.22 | 0.32 | 0.62 | 1.46 |
| Kabale | 0 | 0 | 5 | 0 | 2 | 1 | 0 | **8** | **3** | 0.96 | 0.90 | 0.53 | 0.50 | 0.50 | 2.46 |
| Tororo | 1 | 0 | 0 | 2 | 0 | 0 | 0 | **3** | **3** | 0.91 | 0.64 | 0.44 | 0.64 | 0.80 | 1.90 |
| Mayuge | 8 | 2 | 0 | 0 | 0 | 0 | 0 | **10** | **2** | 0.43 | 0.50 | 0.32 | 0.46 | 0.70 | 1.65 |
| Isingiro | 0 | 1 | 1 | 3 | 1 | 0 | 1 | **7** | **5** | 2.06 | 1.50 | 0.73 | 0.50 | 0.42 | 4.37 |
| Fort portal | 0 | 0 | 4 | 0 | 0 | 0 | 0 | **4** | **1** | 0.00 | 0.00 | 0.00 | nan | 0.00 | 0.00 |
| Ibanda | 0 | 2 | 2 | 3 | 0 | 0 | 0 | **7** | **3** | 1.03 | 1.10 | 0.70 | 0.60 | 0.64 | 2.94 |
| Luwero | 8 | 4 | 0 | 0 | 0 | 0 | 0 | **12** | **2** | 0.40 | 0.64 | 0.44 | 0.64 | 0.80 | 1.90 |
| Mukono | 6 | 0 | 1 | 0 | 0 | 0 | 0 | **7** | **2** | 0.51 | 0.41 | 0.25 | 0.35 | 0.64 | 1.51 |
| Total number | **26** | **23** | **13** | **8** | **3** | **1** | **1** | **75** |  | | | | | | |
